# The Jun-dependent axon regeneration gene program: Jun promotes regeneration over plasticity

**DOI:** 10.1093/hmg/ddab315

**Published:** 2021-10-28

**Authors:** Matthew R J Mason, Susan van Erp, Kim Wolzak, Axel Behrens, Gennadij Raivich, Joost Verhaagen

**Affiliations:** Laboratory for Regeneration of Sensorimotor Systems, The Netherlands Institute for Neuroscience, Royal Netherlands Academy of Arts and Sciences (KNAW), Meibergdreef 47, Amsterdam 1105BA, The Netherlands; Laboratory for Regeneration of Sensorimotor Systems, The Netherlands Institute for Neuroscience, Royal Netherlands Academy of Arts and Sciences (KNAW), Meibergdreef 47, Amsterdam 1105BA, The Netherlands; Laboratory for Regeneration of Sensorimotor Systems, The Netherlands Institute for Neuroscience, Royal Netherlands Academy of Arts and Sciences (KNAW), Meibergdreef 47, Amsterdam 1105BA, The Netherlands; Cancer Stem Cell Laboratory, The Institute of Cancer Research, 237 Fulham Road, London SW3 6JB, UK; Department of Surgery and Cancer, Imperial College London, London SW7 2AZ, UK; Convergence Science Centre, Imperial College, London, SW7 2BU, UK; UCL Institute for Women's Health, Maternal and Fetal Medicine, Perinatal Brain Repair Group, London WC1E 6HX, UK; Laboratory for Regeneration of Sensorimotor Systems, The Netherlands Institute for Neuroscience, Royal Netherlands Academy of Arts and Sciences (KNAW), Meibergdreef 47, Amsterdam 1105BA, The Netherlands; Center for Neurogenomics and Cognition Research, Neuroscience Campus Amsterdam, Vrije Universiteit Amsterdam, Amsterdam 1081HV, The Netherlands

## Abstract

The regeneration-associated gene (RAG) expression program is activated in injured peripheral neurons after axotomy and enables long-distance axon re-growth. Over 1000 genes are regulated, and many transcription factors are upregulated or activated as part of this response. However, a detailed picture of how RAG expression is regulated is lacking. In particular, the transcriptional targets and specific functions of the various transcription factors are unclear. Jun was the first-regeneration-associated transcription factor identified and the first shown to be functionally important. Here we fully define the role of Jun in the RAG expression program in regenerating facial motor neurons. At 1, 4 and 14 days after axotomy, Jun upregulates 11, 23 and 44% of the RAG program, respectively. Jun functions relevant to regeneration include cytoskeleton production, metabolic functions and cell activation, and the downregulation of neurotransmission machinery. *In silico* analysis of promoter regions of Jun targets identifies stronger over-representation of AP1-like sites than CRE-like sites, although CRE sites were also over-represented in regions flanking AP1 sites. Strikingly, in motor neurons lacking Jun, an alternative SRF-dependent gene expression program is initiated after axotomy. The promoters of these newly expressed genes exhibit over-representation of CRE sites in regions near to SRF target sites. This alternative gene expression program includes plasticity-associated transcription factors and leads to an aberrant early increase in synapse density on motor neurons. Jun thus has the important function in the early phase after axotomy of pushing the injured neuron away from a plasticity response and towards a regenerative phenotype.

## Introduction

Neurons axotomized by peripheral nerve injury initiate a gene expression program, which not only facilitates long-distance regeneration in peripheral nerves, but can also achieve growth in inhibitory areas such as sites of spinal cord injury (1). A strong neuron-intrinsic regeneration response is likely to be required to promote regeneration of central nervous system neurons (2,3). Understanding how regeneration-associated genes (RAGs) are regulated is thus an important step towards the therapeutic goal of the artificial induction of the RAG program. RAG expression appears to be triggered after peripheral axotomy by a combination of retrograde signals and calcium signaling, leading to the upregulation and/or activation of a considerable number of transcription factors (TFs), including ATF3, Jun, CREB, STAT3, Smad1, CEBPD and Klf family factors (4), and is also accompanied by epigenetic changes, in particular histone acetylation and increased chromatin accessibility (5–7). In general, attempts to activate the RAG program by over-expressing these TFs or activating them by delivering upstream signaling partners have had positive but limited effects in peripheral neurons (8–12) and in corticospinal neurons (13,14).

Thus, although a fairly complete picture of the regulated genes in regenerating neurons has been acquired (15–21), many TFs are known to be involved, and it is still not well understood how this gene expression program is regulated. Although in the case of several TFs a small number of targets have been identified (8,22–24), and indeed an important role for ATF3 in activating regenerative gene transcription was shown in sensory neurons (25), in general, it is not known which RAGs are regulated by which TF and to what degree, and whether key RAG TFs also upregulate other RAG TFs. Little is known about how TFs co-operate in regenerating peripheral neurons. More broadly for most TFs, including Jun, it is not possible to ascribe to them a particular function in the regeneration program.

Jun was the first TF to be identified as regulated during regeneration (26) and the first shown to be functionally important for regeneration (22). A study of the RAG regulatory network identified Jun as one of the key hubs of this network (27). Jun forms dimers with Jun, Fos, ATF and JDP family proteins to create the TF complex known as AP1 (28). Footprinting analysis of regulatory regions with increased accessibility after axotomy also identified many variations of AP1-like binding sites as over-represented (7). However, as we showed previously, over-expression of Jun along with 3 of its binding or hub network partners (ATF3, STAT3 and Smad1) failed to significantly boost regeneration in a CNS injury model and did not exceed the effects of over-expressing ATF3 alone in the more permissive dorsal root injury model (11). A more complete understanding of how Jun regulates RAG expression would therefore be of great benefit.

Jun homo- and heterodimers can act via AP1 sites and CRE sites (29), with the preference varying according to the dimerization partner. During axon regeneration, it is not clear whether Jun acts more via AP1-like sites or CRE-like sites, or both, and in a recent ChipSEQ study of Jun binding sites both types of binding site were bound during regeneration (30).

In this study, we have carried out gene expression profiling by microarray of regenerating mouse facial motor neurons in which Jun was knocked out. We have made a comprehensive determination of the contribution of Jun to successful regeneration in facial motor neurons, in terms of target genes, functions and regulatory mechanisms at the promoter level. We quantify the contribution of Jun to the RAG program and show it is essential for the upregulation of 254 genes. Jun target genes are found in a variety of functional categories. Deep *in silico* analysis of the promoter regions of Jun-dependent RAGs reveals that these promoters display over-representation of AP1 sites, much more than of CRE sites, although these are found more frequently near to AP1 sites. Furthermore, we show that in the absence of Jun the neuronal response to axotomy begins with a plasticity-type response involving an increase in synapses on the motor neurons. In facial motor neurons, Jun thus has a specific function in pushing the neuronal injury response towards regeneration rather than synaptic plasticity.

## Results

### Jun deletion causes profound differences in gene expression after axotomy

**Figure 1:**
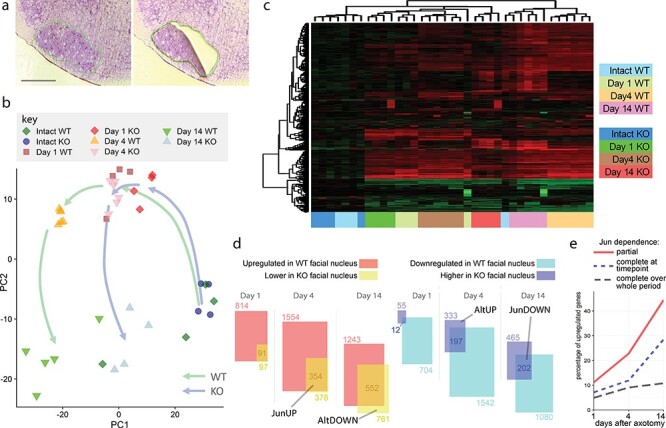
Jun deletion causes profound differences in gene expression after axotomy. (**a**) Cresyl violet-stained facial nucleus (outlined in green) shown before (left) and after laser dissection. Scale bar 500 μm. (**b**) Principle component analysis of WT and KO gene expression profiles 1–14 days after facial nerve injury and in uninjured animals, performed with the 500 most variable genes. Each point represents one animal. While uninjured WT and KO animals cluster together, at day 1 after injury the two genotypes begin to move apart and are clearly separable at day 4 and day 14, while within each time point/genotype animals cluster near each other, apart from one uninjured wild-type animal. The trajectory of KO animals (blue arrow) shows a reduced distance of travel in principle component 1 compared to the WT trajectory (green arrow), reflecting the reduced regulation of Jun target genes. (**c**) Heatmap of gene expression profiles with hierarchical clustering. Genotype and time point are indicated by color on the *x*-axis as shown in the key. In general, animals at the same time point and genotype cluster together. (**d**) Numbers of genes regulated by axotomy and differentially regulated with Jun deletion in facial motor neurons. In the left panel the overlap of the two boxes represents genes upregulated in a Jun-dependent manner (the JunUP class). The AltDOWN class (newly or more strongly downregulated genes in the KO) is also indicated. In the right-panel, the overlapping areas represent Jun-dependent downregulated genes (the JunDOWN class) while the purple area indicates genes that are *de novo* upregulated or more strongly upregulated by axotomy in KO animals (the AltUP class). (**e**) Percentages of regeneration-associated genes that show complete dependence on Jun after axotomy at each time point (so not significantly regulated in KO animals after axotomy, compared to uninjured animals), partial dependence (still upregulated after axotomy in KO mice but significantly less than in WT mice) and complete dependence on Jun over the whole time-course (no residual upregulation seen at any time point).

Floxed Jun mice crossed with nestin-cre mice (referred to as KO), in which Jun is deleted in the central nervous system, and cre-negative littermates (referred to as WT) received a facial nerve injury. Facial motor nuclei, visualized by cresyl violet staining (Fig. 1a), were excised by laser dissection from a total of 29 animals after 1 day, 4 days or 14 days after facial nerve injury or after no injury. RNA was extracted and gene expression was performed by means of microarray.

Principle component analysis of the 500 most variable genes (Fig. 1b), and hierarchical clustering of samples (Fig. 1c) showed that, with the exception of one WT animal, the uninjured WT and KO animals clustered together. At day one, separation between the genotypes is already visible and is clear at days 4 and 14, with the main difference being an obviously reduced change in principal component 1. The trajectories of the expression profiles of the two genotypes are indicated by arrows in Figure 1b. Expression of the cell-type specific marker genes GFAP, Aldh1h, Tubb3, Eno2 and Mobp in intact animals were similar (Supplementary Material, Fig. S1), suggesting that the overall contribution of different cell types to the RNA was not substantially different between genotypes. For a number of selected genes the gene expression changes were verified by quantitative RT-PCR with the RNA samples used for microarray analysis (Supplementary Material, Fig. S2). In almost all cases, the expression profiles generated by qPCR very closely reflected those generated by microarray.

### Jun both induces and suppresses gene regulation in both directions

Jun deletion had little effect on gene expression prior to axotomy; only 25 genes (other than Jun) were differentially expressed between WT and KOs uninjured animals (listed in Supplementary Material, Dataset 1). Jun deletion resulted in both reduced up- and downregulation of axotomy-responsive genes, as well as increased regulation of some genes in both directions, and *de novo* regulation of other genes (defined as up- or downregulation of genes not regulated after axotomy in WT animals). Differentially expressed genes were thus categorized into four classes at each time point, based on the regulation induced by axotomy in wild type animals and the effect of Jun deletion on expression (see section Materials and Methods and Table 1). The JunUP class, indicated in Figure 1d, contains RAGs with Jun-dependent upregulation after axotomy, genes which are likely to be transcriptional targets of Jun. JunUP genes grew as a proportion of RAGs from 11% at day 1 to 44% at day 14 (Fig. 1d and e). Approximately half the JunUP genes at each time point are completely dependent on Jun for their upregulation, meaning that they are not regulated at all by axotomy in the absence of Jun (Fig. 1e), with the remainder defined as partially dependent (still regulated by axotomy in KO animals but significantly less than in WT mice). Over the whole time course, 254 genes, or about 10% of the RAG program, were in the JunUP category at some time point and not regulated in the knockouts at any time point (Fig. 1e). The full classification of differentially expressed genes between WT and KO animals at each time point is given in Supplementary Material, Dataset 1.

**Table 1:** Classification scheme for genes that were differentially expressed between WT and KO animals. For each time point, genes were first classified according to wild-type regulation after axotomy, compared to uninjured animals, and then according to the differential expression between WT and KO animals

Regulation in WT following axotomy	Relative expression in KO compared to WT	Regulatory class	Description
Up	Lower	JunUP	Jun-upregulated
Down	Higher	JunDOWN	Jun-downregulated
Up	Higher	AltUP	Alternative upregulated
No change	Higher		
Down	Lower	AltDOWN	Alternative repressed
No change	Lower		

The JunDOWN class (see Fig. 1d) contains genes showing Jun-dependent downregulation after axotomy. Jun downregulates relatively fewer genes than it upregulates (growing from 2% of downregulated genes at 1 day to 19% at day 14). Also indicated in Figure 1d are the AltUP class, containing genes, which are *de novo* upregulated after axotomy and RAGs with increased upregulation in the KO; and the small AltDOWN class, genes which are newly downregulated or with increased downregulation in the KO.

### Jun regulates a subset of classical RAGs and many previously unidentified RAGs

**Figure 2:**
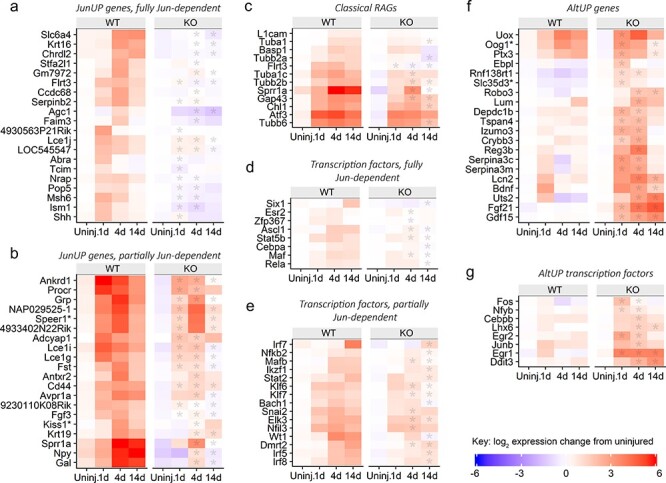
Heatmaps depicting differential gene expression for selected genes in WT and KO facial motor nuclei after axotomy, showing that Jun regulates a subset of RAGs, including several transcription factors and suppresses an alternative gene expression program. (**a**) Genes which show Jun-dependent upregulation after axotomy (the JunUP regulatory class), at both 1 day and 4 days (and in some cases also at 14 days) with no significant upregulation in KO animals. (**b**) Genes in JunUP where upregulation is partially Jun-dependent at 1 day or 4 days. In many cases, at 14 days upregulation continues in WT animals but returns to baseline in the KO. (**c**) Well-known regeneration-associated genes (RAGs) show a range of effects of Jun deletion. Gap43, Sprr1a and several tubulins (Tuba1c, Tubb2b) are lower from 4 days onwards. Other RAGs (Atf3, Basp1) are not significantly affected by Jun expression. (**d**) Transcription factors in JunUP with no regulation in KO animals. (**e**) Transcription factors in JunUP, which do still show significant regulation in KO animals. (**f**) Genes which show *de novo* or increased upregulation after axotomy in KO motor neurons (the AltUP regulatory class). The 20 most differentially expressed genes are shown. This appears to be a gene expression program that is induced by axotomy but normally suppressed by Jun. Some genes, such as Fgf21 and Gdf15, show strong and sustained upregulation. (**g**) Transcription factors in AltUP. Early upregulation of plasticity-associated TFs Fos, Egr1 and Egr2 is seen. For all genes shown, no significant expression difference between genotypes in intact animals was detected. Asterisks indicate a significant difference in expression between WT and KO animals (FDR <0.01, fold-change >1.5). See Supplementary Material, Figure S2 for qPCR validation of expression profiles.

RAGs with complete dependence on Jun over days 1–4 are shown in gene expression heatmaps in Figure 2a, while genes with partial dependence are shown in Figure 2b. Some known RAGs are found in each category (e.g. Flrt3 is fully Jun-dependent in Figure 2a and Sprr1a partially so in Fig. 2b). Many novel RAGs with strong Jun-dependent upregulation are also seen (e.g. Pop5, Ccdc68, Lce1i, Speer1 and a long non-coding RNA, 9230110K08Rik).

Several additional classical or well-known RAGs showed a range of effects as shown in Figure 2c, with a partial dependence on Jun being the most common signature (e.g. GAP43 and the tubulin isoforms Tubb2b and Tuba1c). Five neuropeptide genes were upregulated (more than 10-fold) and were Jun-dependent to varying degrees (Supplementary Material, Figure S3a).

### Jun controls expression of a subset of other regeneration-associated transcription factors (RAG TFs)

Expression profiles of RAG TFs are of interest because they indicate how Jun may be positioned relatively in the hierarchy of the gene regulatory network. At day 1, no TFs showed dependency on Jun for their upregulation. Eight TFs were fully dependent on Jun at later time points: Ascl1, Cebpa, Esr2, Maf, Stat5b, Zfp367 (Fig. 2d), and, at 14 days only, Rela and Six1. At day 4, a further seven TFs were upregulated in a partially Jun-dependent manner, including the known RAG TFs Klf6 and Klf7, plus Dmrt2, Elk3, Mafb, Nfil3 and Snai2 (Fig. 2e) and eight more Jun-dependent TFs at 14 days. Two known RAG TFs, JunB and Cebpb, were also upregulated by axotomy but were in fact more highly expressed in mutant animals and thus belong in the AltUP class. Other well-known RAG TFs (e.g. Atf3, Stat3, Smad1) were not significantly affected by Jun deletion (examples are shown in Supplementary Material, Fig. S3b). Six additional AP1 family members were upregulated by axotomy: Batf3, Batf, Fosl1, Fosl2, Atf4 and Atf5, but these were mostly unaffected by Jun deletion (Supplementary Material, Fig. S3c). Thus, Jun commands a segment (approximately 10%) of the regeneration-associated transcription factor response from day 4. A full list of differentially expressed Jun-dependent TFs is given in Supplementary Material, Dataset 1.

### An alternative gene upregulation program takes place in the KO

Strikingly, in the absence of Jun, a large number of genes were upregulated that are not upregulated in WT animals, and some RAGs showed increased upregulation instead of decreased expression. Together, these genes form the AltUP class, indicated in Figure 1d. Thus, it appears that an alternative gene expression program is triggered by axotomy in the absence of Jun. Expression heatmaps of some of these genes are shown in Figure 2f-g. Notably, a number of TFs are members of this class, including Fos, Egr1, Egr2 and Ddit3 (Fig. 2g).

### Functions of Jun in regeneration

**Figure 3:**
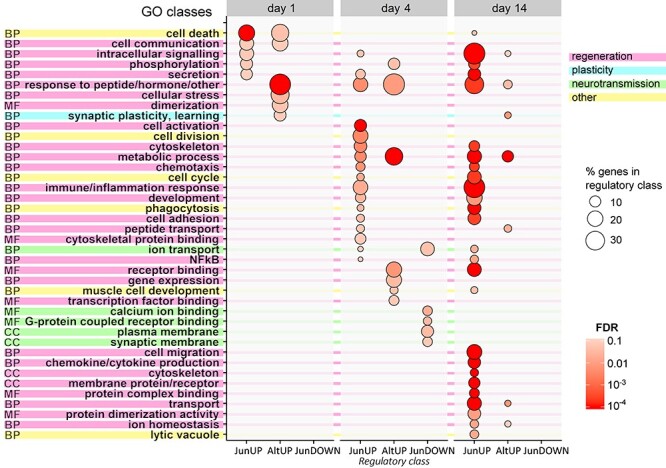
GO analysis of the JunUP, AltUP and JunDOWN regulatory classes shows that most functions of Jun-dependent genes after axotomy are relevant to regeneration. Shown is an overview of broad categories of over-represented GO classes. Circle fill color indicates FDR of the most significant GO class per category. Size represents percentage of genes in the regulatory class, as shown in the key (only categories with at least 5% of genes are shown). Categories are color-coded to distinguish regeneration-relevant functions, other functions, Jun-downregulated neurotransmission-related classes and the Jun-suppressed plasticity response. Initially (at day 1), the range of over-represented functions is small, but these become broader with time as many more processes are affected at day 4 and day 14. Known functions of Jun such as cell-death and control of cell cycle are represented but are outnumbered by classes related to regeneration, such as cytoskeleton organization, cell adhesion and intracellular signaling. Together with cell activation and metabolic functions, these suggest an overall activation of cellular metabolism and cytoskeleton production as major pro-regenerative functions of Jun. Meanwhile, Jun also downregulates genes related to neurotransmission (in the JunDOWN class), and AltUP genes contain synaptic plasticity classes as well as metabolic functions. See Figs S3 and S4 for expression profiles of genes by functional category.

To understand more about the targets of Jun in regeneration, we performed Gene Ontology (GO) over-representation analysis on the genes in the JunUP, JunDOWN and AltUP classes. Over-represented GO classes were grouped into broader categories for summarization (Fig. 3) (The full analysis is available in Supplementary Material, Dataset 2.)

In the JunUP class, most identified categories of function were regeneration-related activities (labeled pink in Fig. 3), a number of which have not previously been ascribed to Jun. The remaining functions (labeled yellow in Fig. 3) include well-known activities of Jun in other cell types, such as cell-death and cell cycle/cell division. At day 1, classes related to cell death were the most significant (expression profiles are shown in Supplementary Material, Fig. S4a), along with both extracellular and intracellular signaling, and classes related to secretion. At day 4, classes related to cell cycle and cell division (see Supplementary Material, Fig. S4b) were among the most significant. However, the most significant class overall was cell activation, while classes relevant to regeneration, such as cytoskeleton organization (including several myosins; Supplementary Material, Fig. S4c) and cell adhesion, were also over-represented. Metabolic functions also feature prominently. In general, at 4 days, JunUP targets appear to contribute to a general activation of the cell, increased metabolism and structural components of the regenerating axon. At day 14, additional relevant classes such as cell migration and transport appear. Immune and inflammatory processes also become prominent, likely due to the greater influx of T-cells in WT animals (22) at this time point. The genes in cell death-related GO classes, such as Casp6, Msh6, Hax1 and Igfbp3, are all previously unidentified pro-apoptotic targets of Jun and do not include the already reported pro-apoptotic Jun targets, Hrk (31) and Bcl2l11 (32). Similarly, although control of the cell cycle is a known Jun function (33), the JunUP genes with this function are newly identified targets, e.g. the cyclins Ccna2, Ccnb1, Ccnb2 and the cyclin-dependent kinase Cdk6.

### Jun downregulates genes for neurotransmission

In the JunDOWN set of genes (Jun-dependent axotomy-downregulated genes), over-representation of genes related to neurotransmission was seen at 4 days (labeled green in Fig. 3). Specifically, members of the GO classes for voltage-gated potassium channels and post-synaptic cell components (Supplementary Material, Fig. S4d and e) were all downregulated in a Jun-dependent manner. Jun thus appears to control a reduction in neurotransmission machinery after axotomy.

### Jun suppresses a gene expression response directed at synaptic plasticity

The AltUP program represents a gene expression response to axotomy that is usually suppressed by Jun. GO analysis of the early AltUP program (genes induced by axotomy when Jun is absent) revealed GO classes related to synaptic plasticity and learning and memory (labeled blue in Fig. 3). In the day 1 AltUP program, these classes included the TFs Fos, Egr1 and Egr2, and several other rapidly upregulated plasticity-associated genes, shown in Supplementary Material, Figure S4f. The AltUP class also contained some categories of over-represented GO classes similar to those found in the JunUP group (even though the two groups contain no shared genes, by definition), such as cell death and cell communication at day 1, and numerous metabolic processes at days 4 and 14, suggesting compensatory changes that take place in response to Jun deletion and the resultant lack of certain (particularly metabolic) functions.

### Histochemical validation shows JunUP and AltUP genes are regulated specifically in neurons

**Figure 4:**
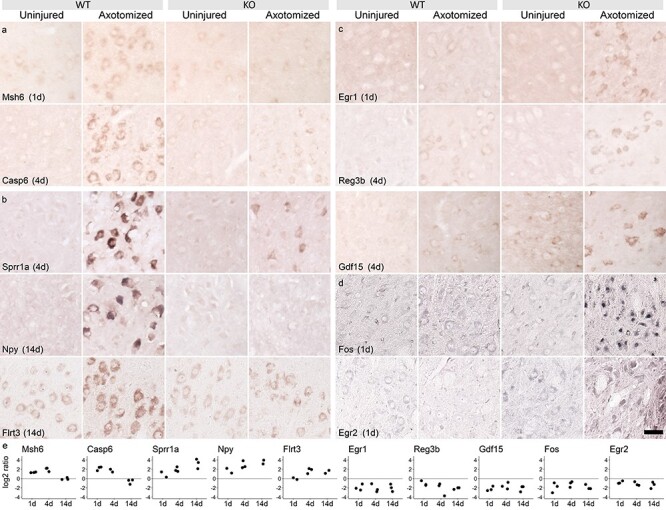
JunUP and AltUP genes are regulated specifically in neurons. (**a**-**c**) ISH and (**d**) immunohistochemistry (IHC). Time points after axotomy are indicated by the labels. (a) ISH for two apoptosis-linked Jun targets, Msh6 and Casp6. (b) ISH for three known regeneration-associated genes, Sprr1a, Npy and Flrt3, with strong Jun dependence. (c) ISH for three genes in the AltUP category, Egr1, Reg3b and Gdf15. (d) IHC for two genes in the AltUP category at day 1, namely Fos and Egr2. All targets are regulated as expected and upregulation is confined to the facial motor neurons. Scale bar 50 μm. See Supplementary Material, Figure S5 for further examples. (**e**) Quantification of gene and protein expression differences in histological stainings between WT and KO animals. Graphs show the ratio of WT to KO staining intensities in cytoplasm (ISH) and nuclei (IHC for Fos and Egr2) and confirm the differential expression profiles after axotomy in facial motor neurons between WT and KO animals.

The nestin-Cre mouse line used here should delete Jun in all cells of the facial motor nucleus and not just motor neurons. Jun expression is confined to motor neurons after axotomy (34), so the effects on gene expression are expected to be neuronal. Nonetheless, we sought to confirm that most gene expression changes we detected were in motor neurons. We carried out *in situ* hybridization (ISH) or immunohistochemistry for a number of genes in the JunUP and AltUP groups. Targets for validation were selected that showed clear differential expression between WT and KO animals. Targets in Figure 4 were selected to represent the following different facets of Jun function: apoptosis, well-known RAGs, and the AltUP program.

All selected targets were upregulated specifically in motor neurons, in a manner consistent with the gene expression profiling. Figure 4a shows ISH for two apoptosis related genes, Msh6 and Casp6, while Figure 4b shows ISH for Sprr1a, Npy and Flrt3, all known RAGs that are Jun dependent. Figure 4c shows ISH for Egr1, Reg3b and Gdf15, all genes that take part in the AltUP program, while Figure 4d shows immunohistochemistry for two TFs in the AltUP program: Fos and Egr2. These AltUP genes can also be seen to be upregulated specifically in motor neurons. Validation was carried out at all time points in multiple animals (*n* = 2–3). The time point shown for each gene was chosen where the differential regulation between genotypes was the strongest according to the gene expression profiles. Quantification of gene expression and protein expression differences observed by ISH and immunohistochemistry, expressed as the ratio of intensities in facial motor neurons in WT and KO sections, is shown in Figure 4e, and confirms the differential expression of these genes after axotomy over the time course in facial motor neurons.

Lastly, Supplementary Material, Figure S5 shows seven other genes, selected to represent the following functional categories: cytoskeleton, novel RAGs, apoptosis, cell signaling and cell division. These genes are as follows: Ccdc68, Nrap (cytoskeletal functions); Speer3, Pop5 (novel RAGs); Rras2 (cell death); Shh (cell signaling); and Fgf3 (cell division). Again, these genes were all upregulated specifically in motor neurons, and in a manner consistent with the gene expression profiling.

**Figure 5:**
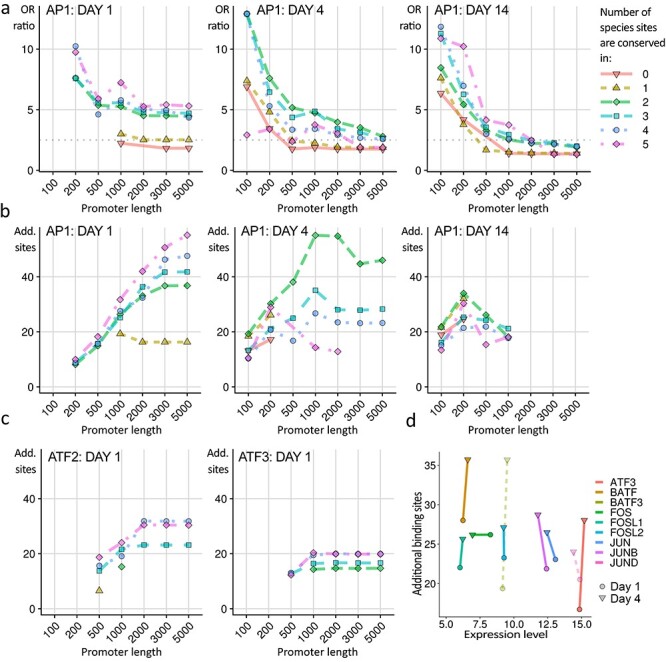
Promoter analysis reveals predominant over-representation of AP1 sites in Jun-dependent genes. (**a**) Over-representation (OR) ratio (of sites in regulated promoters to sites in an unregulated promoter set) of AP1 sites in JunUP promoters at each time point was calculated for varying promoter lengths and requirements for cross-species conservation, using the AP1 position weight matrix in Supplementary Material, Figure S6. The requirement for conservation of binding site scores across additional species dramatically improves the OR ratios, and is essential to detect OR ratios above 2.5 (indicated by a dotted line) in most cases. Short promoter sequences give higher ratios, likely because of clustering of AP1 sites near transcription start sites. Here, all over-representation is significant at *P* < 0.005 (binomial test; FDR <0.01). Conservation in rat, guinea pig, rabbit, human and marmoset was included. (**b**) As an alternative measure to the ratio of sites, we calculated the number of additional sites (‘AS’) compared to that expected from the control promoter set. Optimizing the AS score identifies more binding sites than optimizing for ratio. AS increases with promoter length (shown on the *x*-axis), unlike OR ratio. AS also benefits from the requirement for conservation. Here, a minimum ratio threshold of 2.5 was imposed. (**c**) Over-representation of CRE sites in JunUP promoters, optimized for AS score. ATF2 and ATF3 binding sites (both CRE-like) were only over-represented at day 1, and less robustly so than AP1 sites. Results for CREBP1 sites were similar to ATF3 (not shown). (**d**) Promoter analysis of binding sites for Jun dimers with various partners. AS score is plotted against expression level, for day 1 (circle) and day 4 (triangle) for position weight matrices representing JUN dimerized with the factor indicated. JUN:JUND and JUN:BATF3 matrices were not available, so JUND and BATF3 homodimers are shown instead with dotted lines. The data are consistent with regulation by Jun dimers with ATF3, AP1 family members including JUNB and FOSL2, but strong signals are seen for the two related factors BATF and BATF3.

### *In silico* analysis of promoter regions identifies much greater over-representation of AP1 sites than CRE sites

In order to learn more about possible mechanisms of Jun-dependent RAG regulation, we performed an *in silico* analysis of sequences up to 5 kb upstream of the transcription start sites (TSS). These sequences include the core promoters (35–50 bp upstream of the TSS), the proximal promoters (up to 250 bp upstream) and distal promoter elements (over 250 bp upstream). We refer to the sequences up to 5 kb upstream of the TSS as ‘promoter regions’. These regions are important sites of transcriptional regulation. Although regulation also takes place outside these regions in distal enhancers and also in downstream sequences, analysis of these regions can shed light on the subset of regulatory mechanisms that take place in these areas. We sought evidence for the presence of AP1-like or CRE-like sites in the promoter regions of Jun-dependent RAGs, and we aimed, where possible, to determine if motifs corresponding to particular dimerization partners were preferred. We also looked for binding site motifs present in promoter regions of *de novo* upregulated genes in the AltUP class. Sequence motifs of typical AP1 and CRE sites are shown for comparison in Supplementary Material, Figure S6a. Note that CRE sites are highly similar to AP1, with the addition of a single base in the motif center.

We looked for over-representation (OR) of target sites in promoters of interest compared to a control set of promoters of unregulated genes. We found that using a flexible scoring threshold (explained in section Materials and Methods) improved sensitivity. The efficacy of this approach is demonstrated in Suppl. Fig. S6b, where over-representation of an AP1 site is tested in the day 1 JunUP promoters and the day 4 JunUP promoters, compared to an unregulated promoter set, at a range of scoring thresholds. At day 1, OR is significant at a range of thresholds below 88%, whereas at day 4, OR is achieved at a set of thresholds mostly above 88%, and the two sets of thresholds are nearly mutually exclusive. With a fixed threshold, OR of AP1 sites in either or both promoter sets would likely not be found. The flexible threshold therefore ensures superior sensitivity compared to a fixed threshold approach.

We also incorporated a requirement for conservation across multiple species (conservation in rat, guinea pig, rabbit, human and marmoset was considered) (see section Materials and Methods). We searched for over-representation of AP1 sites and of ATF2, ATF3 and CREB1 binding sites (all CRE-like), optimizing first for over-representation ratio and then for ‘Additional Sites’ (AS) i.e. the increase in number of sites found over that expected based on the control promoters. We searched exhaustively, varying conservation (in 0–5 species besides mouse) and promoter length. AP1 site over-representation was found at all time points in the JunUP promoters (Fig. 5a and b), while CRE-like sites were less strongly over-represented and only found at day 1 (Fig. 5c). Imposing a requirement for conservation across species markedly improved the signal of both binding sites. For subsequent analyses, a promoter length of 1 kb and conservation in mouse plus three species was chosen, as this gave clear signals for both types of binding site.

**Figure 6:**
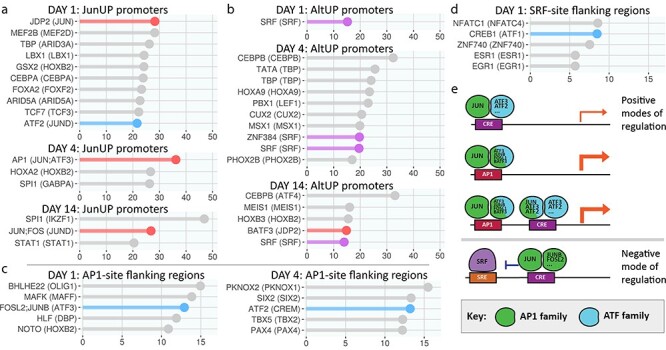
Further analysis of JunUP promoters and AltUP promoters, leading to proposed modes of regulation by Jun. (**a**-**c**) Using 1 kb promoters, requirement for conservation in three additional species besides mouse, and optimizing for the additional sites (AS) score, we analyzed the JunUP promoters for all binding sites in our database. Often, multiple position weight matrices (PWMs) corresponding to different TFs detect sites at the same locations, so PWMs detecting hits at the same locations were grouped together. The PWM with highest AS score is indicated first. In brackets is the highest-expressed TF in the same location group with at least 75% of the highest score. AP1 sites (red bars) show highest over-representation scores at day 1 and day 4, and second highest at day 14. CRE sites were 10th highest at day 1. (b) Analysis of AltUP promoters shows SRF sites as the only type over-represented at day 1, while at later time points CEBP sites dominate. (c) We analyzed the flanking regions of AP1 sites found in (a) to identify possible co-operating factors. Here, we found CRE sites (blue bars) over-represented in these flanking regions. (**d**) Flanking region analysis of SRF sites found in (b) identifies CRE sites (blue bar) are over-represented in these regions. (**e**) Four proposed modes of transcriptional regulation by Jun suggested by the analysis in (a-e). Jun increases transcription predominantly via AP1 sites, and at AP1 and CRE sites in proximity, but only weakly from CRE sites alone. Meanwhile, Jun blocks SRF activity via CRE sites in close proximity to SRF binding sites.

We examined AS scores for Jun dimers with specific partners (Fig. 5d), and plotted these against expression level. Our analysis is compatible with target gene regulation by Jun dimerizing with ATF3, as well as JUNB, FOSL2 and other AP1 members, but strong signals were also found for BATF and BATF3.

We then tested all binding site matrices for the AS measure in JunUP promoters (Fig. 6a). At day 1 and day 4, AP1 sites are the most strongly over-represented sites and are second highest at day 14, while the only appearance of CRE sites is at day 1, as 10th highest.

We also analyzed the promoters of the AltUP genes. We found over-representation of binding sites for serum response factor (SRF) in these promoters. At day 1, this was the only site showing significant over-representation (*P* = 10^−7^, binomial test; false discovery rate (FDR) = 0.24). At day 4 and day 14, SRF sites were still over-represented, but CEBP family sites showed the greatest AS score (Fig. 6b).

To identify factors potentially co-operating with Jun in transcriptional regulation, we examined flanking regions (100 bp on either side) of AP1 sites in JunUP promoters, and of SRF sites in AltUP promoters. At day 1 and day 4 we found over-representation of CRE sites near AP1 sites (Fig. 6c). Lastly, we found CRE sites overrepresented near SRF binding sites in day 1 AltUP promoters (Fig. 6d). These *in silico* findings lead us to hypothesize that, in promoter regions, Jun acts via the four modes of transcriptional regulation shown in Figure 6e, where Jun-induced upregulation is predominantly via AP1 sites, and also via AP1 and CRE sites in close proximity, but only weakly from CRE sites by themselves, while Jun suppression of the AltUP program is via CRE sites near to SRF sites.

### The aberrant plasticity response, usually suppressed by Jun, manifests as an increase in synaptic density

GO analysis of the AltUP program, containing genes usually suppressed by Jun, revealed GO classes related to synaptic plasticity, learning and cognition. We found SRF binding sites over-represented in AltUP promoters, and indeed AltUP members Fos, Egr1 and Egr2 are known targets of SRF (35). Furthermore all four TFs are linked to regulation of synaptic plasticity and learning (36–39).

The synaptic plasticity functional class included three out of eight TFs in the AltUP class, and Egr1 in particular was one of the most strongly upregulated genes. Several other strongly upregulated genes (Fgf21, Gdf15) may affect synaptic plasticity (40,41) and are SRF targets (42,43). Together, these data indicate that a synaptic plasticity response is a major component of the AltUP program. Thus, it appears that Jun suppresses a plasticity program in facial motor neurons that can nonetheless be triggered by axotomy when Jun is absent.

The existence of a plasticity response in axotomized facial motor neurons is of interest because neuronal plasticity, rather than regeneration, is characteristic of the CNS response to injury (e.g. stroke or traumatic brain injury). Motor neurons are located in the CNS but normally respond with a regeneration response following injury to their axons. The existence of this plasticity response in KO facial motor neurons is significant because it suggests Jun has evolved a specific role to favor regeneration in these motor neurons. We therefore chose to study this unexpected response further.

We first investigated whether increased plasticity was detectable in the axotomized facial nuclei in Jun mutants, by quantifying synapse density on motor neuron profiles and on perineuronal dendrites.

**Figure 7:**
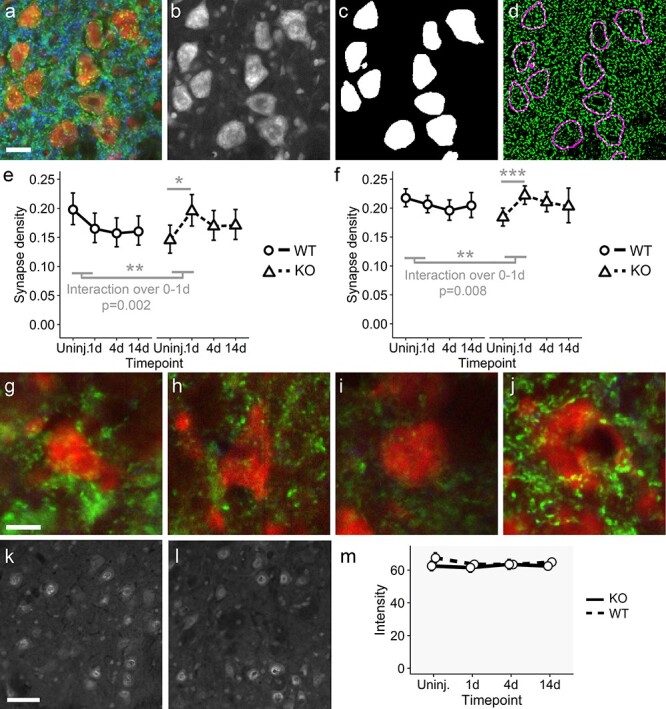
The aberrant plasticity gene expression response, usually suppressed by Jun, manifests as an increase in synaptic density. (**a**) Immunohistochemistry for motor neuron cell bodies (Nissl stain; red), synaptophysin to visualize synapses (green) and MAP2 to label dendrites (blue). (**b**) Single channel image of the Nissl stain. (**c**) Mask generated by the U-NET neural network trained to recognize facial motor neurons. (**d**) Rim of motor neurons identified in (c) overlaid (in magenta) on a thresholded image of the synaptophysin staining. Synapse density was calculated as fraction of green pixels in the neuronal rim area. Scale bar: 20 μm. (**e**) Synapse density changes on motor neuron cell bodies after axotomy in WT and KO mice. In WT animals, synaptic stripping can be observed beginning at day 1. Synapse density remains lower over the time course. (The decline over the whole time course is significant, *P* = 0.04.) ln KO animals, on the other hand, synapse density is lower than in WT animals but increases significantly in the first day (*P* = 0.01), consistent with the observed expression of a plasticity related set of genes in these animals (Fos, Egr1, Egr2). The changes in density over the first day are significantly different between genotypes (*P* = 0.002). (**f**) Synapse density changes on motor neuron dendrites after axotomy in WT and KO mice. Again, synapse density declines slightly on perineuronal dendrites (n.s.). In KO animals, as on the cell bodies, a marked increase is seen in the first day (*P* = 7 × 10^−5^). The changes in density over the first day are significantly different between genotypes (*P* = 0.008). Data in (e) and (f) are shown as mean ± 95% confidence intervals. (**g**-**j**). Synaptophysin staining (green) and Nissl stain (red) shows synapse density changes in WT and KO mice over the first day. Rim synapse densities of the neurons shown are close to the mean values depicted in panel (e). A decrease in density is visible in WT mice from uninjured (g) to day 1 after axotomy (h), while the low initial density in uninjured KO animals (i) and sharp increase 1 day after axotomy (j) are visible. Scale bar 10 μm. (k, l). Immunohistochemistry for SRF in WT (k) and KO (l) facial nuclei 1 day after axotomy. SRF staining intensity was similar between genotypes. Scale bar: 50 μm. (m) Quantification of nuclear SRF labeling at all time points. No difference between genotypes and no significant regulation of SRF protein after axotomy was seen. Data are shown as mean ± SEM. Thus, the expression of SRF target genes in KO mice is not due to higher SRF expression in these animals.

Axotomized (1 day post-injury) and uninjured facial nuclei were immunostained for synaptophysin and MAP2, along with a fluorescent Nissl stain to identify motor neurons. Motor neuron profiles were identified using the neural network U-NET (44) via its ImageJ plug-in, trained on manually segmented images. Synapse density was quantified on cell body profile boundaries (Fig. 7a-d) and on MAP2-stained dendrites within 50 μm of cell bodies.

Surprisingly, in Jun KO motor neurons, the baseline synaptic density was significantly lower than in WT mice (*P* < 0.05). In wild type mice, synaptic density appears to decrease, consistent with the known phenomenon of synaptic stripping (45,46) (Fig. 7e). The decline over the 14 day period was significant (*P* = 0.04; interaction of synaptic density with time over whole time course). Synaptic density decreased in the first day after axotomy and then remained stable, although this initial decrease is not significant. Strikingly, in KO motor neurons a significant increase in synaptic density was observed on cell bodies at day 1 (*P* = 0.01; Fig. 7e), consistent with the idea that Jun-negative motor neurons initially activate a plasticity response. Thus synapse densities in WT and KO show opposite responses in the first day after axotomy, and the time course of synapse density on motor neuron cell bodies over the first day was significantly different between WT and KO animals (interaction of time with genotype, *P* = 0.002).

Synapse density on dendrites close to motor neuron cell bodies showed a similar pattern. Synapse density on dendrites increased significantly (*P* = 7 × 10^−5^) after axotomy (Fig. 7f), while in WT animals, a slight decline in density was seen (n.s.). Again, the time course of synapse density on proximal dendrites over the first day was significantly different between WT and KO animals (interaction of time with genotype, *P* = 0.008). The change in synaptic density can be seen in the images of motor neurons shown in Figure 7g-j. Each neuron shown has a synaptic density close to the means depicted in Figure 7e.

SRF is constitutively expressed in mouse facial motor neurons, but SRF mRNA levels are unaffected by axotomy (Supplementary Material, Fig. S2). No difference was found in SRF protein expression between WT and KO animals (Fig. 7k, l, m). If anything, SRF protein expression was marginally lower in KO animals (n.s.).

### The synaptic density increase is driven by SRF target genes

Fos, Egr1 and Egr2 are well known targets of SRF, and indeed SRF binding sites were heavily over-represented in the AltUP promoters. We therefore hypothesized that blocking expression of SRF target genes after axotomy would substantially block the AltUP program and the accompanying plasticity response. We therefore investigated the effect of expressing a dominant-negative SRF (dnSRF) construct in the KO facial nucleus, using adeno-associated viral (AAV) vectors. A comparison of AAV serotypes revealed that AAV6 gave robust neuron-specific transduction in the mouse facial nucleus (Supplementary Material, Fig. S7). AAV6 vectors containing a dual vector construct expressing dnSRF and fGFP (47), or just fGFP, were injected into the facial nucleus. After 2 weeks, animals were given a facial axotomy and sacrificed after 1 more day, or were sacrificed uninjured.

**Figure 8:**
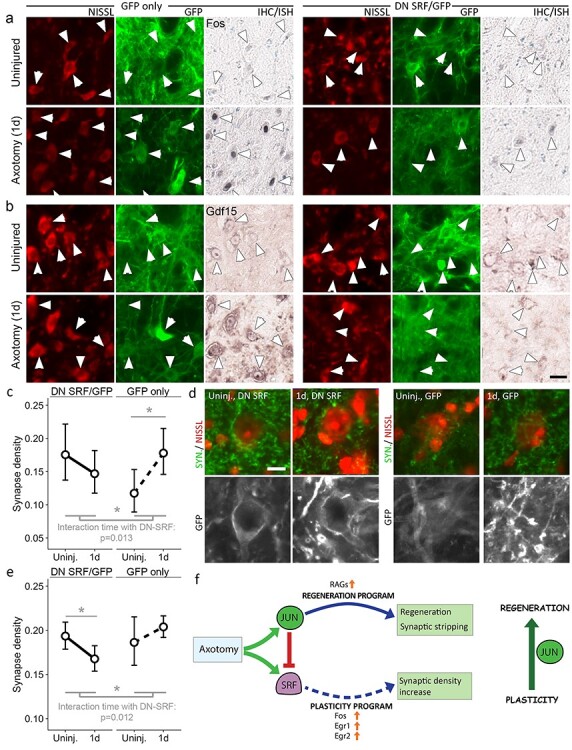
Synaptic density increase is driven by SRF target genes. (**a**-**d**) Dominant negative SRF blocks AltUP gene expression and restores normal synapse density responses. AAV6 vectors expressing both dominant negative SRF (dnSRF) and farnesylated GFP (fGFP), or control vectors expressing fGFP only, were delivered to the facial nucleus in KO mice. See Supplementary Material, Figure S7 for AAV serotype testing. (a, b). Immunohistochemistry for Fos (a) and ISH for Gdf15 (b) in KO animals in neurons expressing fGFP and dnSRF, or fGFP only. The left sets of six panels show Fos and Gdf15 induction in motor neurons expressing fGFP only, while this is blocked in neurons expressing dnSRF (right sets of six panels). Arrows indicate motor neurons visible in Nissl staining that are GFP positive and thus transduced. Scale bar 25 μm. See Supplementary Material, Figure S8 for more examples. (c) DnSRF restores normal synapse density responses. Synapse density was quantified in GFP positive motor neurons and GFP-positive dendrites 1 day after facial nerve injury and in uninjured animals. Baseline synapse density on motor neurons expressing GFP and thus dnSRF is restored to WT levels and decreases slightly (n.s.) as in WT animals, while motor neurons expressing only fGFP show an increase in synapse density, similar to that seen in Figure 7e. Data are shown as mean ± 95% confidence intervals. (d) Synaptophysin (green) and Nissl stain to identify motor neurons (red) (top row). Each motor neuron is GFP-positive and thus transduced (bottom row). The synapse density changes quantified in (c) can be seen. Each motor neuron shown has a rim synapse density close to the mean value shown in (c) for the condition indicated. Scale bar 10 μm. (**e**) On dendrites, while the increase in synapse density on dendrites of fGFP-only expressing neurons is not significant, dnSRF induces a significant decrease, indicating that synaptic stripping is restored. Data are shown as mean ± 95% confidence intervals. (**f**) Schematic depiction of the effect of Jun on neuronal phenotype early after axotomy. In WT animals, axotomy leads to Jun upregulation and activity, leading to RAG upregulation. Synaptic stripping occurs as part of the regeneration program. Axotomy also leads to SRF activation, but SRF activity is normally blocked by Jun (see also Fig. 6e). In the absence of Jun, SRF activity leads to an aberrant plasticity response involving Fos, Egr1 and Egr2 and increased synapse density. Thus, Jun pushes the cell away from a plasticity response towards a regeneration response.

Immunohistochemistry revealed that dnSRF does indeed block expression of Fos (Fig. 8a) and Egr2 (Supplementary Material, Fig. S8a), while ISH shows it also blocks expression of Gdf15 (Fig. 8b), Egr1 and Fgf21 (Supplementary Material, Fig. S8b, c). However, Reg3b upregulation still occurred in dnSRF-expressing neurons (Supplementary Material, Fig. S8d), indicating that other pathways are also active in the AltUP program after axotomy.

Synaptic density measurements in uninjured animals and at 1 day after axotomy were performed on GFP-positive motor neurons and on GFP-positive perineuronal dendrites. As shown in Figure 8c and d, expression of dnSRF reversed the effect of Jun deletion on synaptic density on motor neuron cell bodies, restoring baseline synaptic density to WT levels and restoring the WT pattern of synaptic density changes over the first day (c.f. Fig. 7e). In neurons transduced with GFP-only expressing virus, cell body synapse density increases (*P* = 0.018; Fig. 8c), while in dnSRF/GFP expressing neurons, it decreases slightly (n.s.; Fig. 8c), as it does in WT animals (Fig. 7e). The interaction between virus transgene and time is significant (*P* = 0.013) indicating the two groups behave differently. Synapse density changes can be seen in the images of GFP-positive motor neurons in Figure 8d, corresponding to the four conditions plotted in Figure 8c.

Synaptic density on dendrites shows a similar response (Fig. 8e), with a significant interaction between virus transgene and time (*P* = 0.013). In the GFP-only virus group, the increase in synaptic density is not significant. However, a significant decrease is seen in the dnSRF group (*P* = 0.006), indicating that synaptic stripping is taking place. These results show that the plasticity response in response to axotomy, resulting in new synapse formation on motor neurons, is driven by SRF target genes.

## Discussion

In this study, we have defined the role of Jun in the regeneration-associated gene program of injured facial motor neurons, identifying its regulatory targets, its biological functions and modes of gene regulation, and revealing its key importance in directing the axotomized facial motor neuron towards a regenerative response over a plasticity response. Currently, while many TFs have been linked to functional effects on axon regeneration (8–10,13,22,24,48), relatively little data are available on what these TFs do in terms of gene regulation. Targets of KLF7 have been identified in CNS neurons (49), but not in peripheral neurons, where regeneration is much more efficient. In a conceptually similar study to this one, gene expression profiling in injured facial motor neurons of ATF3 knockout mice was performed, but only a handful of genes were identified as statistically significant (23), although recently a large number of differentially expressed genes were identified in axotomized ATF3-deleted sensory neurons (25). Thus, currently there is a lack of knowledge about the target genes of each RAG TF, its contribution to the RAG program, and the interplay between RAG TFs.

### The contribution of Jun to the RAG program

We find that Jun contributes to upregulation of a significant fraction of the RAG program in facial motor neurons, increasing over time from about 10% at day 1 to around 40% of the RAG genes at 14 days. Approximately half of these genes are absolutely dependent on Jun for their upregulation in response to axon injury. The initial phase of the response to axotomy appears to proceed by multiple pathways, since the upregulation of most other TFs associated with axotomy appears to be largely unaffected in the first 24 hours, but Jun appears to drive expression of 10–20% of the transcription factors going up after axotomy (about half of these being fully Jun-dependent) at later post-lesion time points. In a gene regulatory network, key TFs at the network hubs drive expression of other TFs, and this appears to be the case for Jun.

Successful regeneration occurs after axotomy in multiple types of neurons, e.g. sensory and autonomic neurons in the peripheral nervous system and spinal motor neurons. It remains to be seen whether Jun deletion has s similar impact on RAG expression in these other regenerating cell types.

### Potential modes of regulation by Jun

We find, by *in silico* analysis, heavier over-representation of AP1 sites than CRE sites in Jun-dependent RAG promoter regions. While such an analysis does not allow firm conclusions to be drawn about regulatory mechanisms, these findings suggest that, in regions upstream of transcription start sites, direct Jun regulation of RAG targets may occur predominantly via AP1 sites rather than CRE sites, although we find evidence for some CRE-dependent targets as well as regulation via adjacent AP1 and CRE sites. This is in contrast to a recent Chip-SEQ analysis of Jun binding-sites in regenerating neurons, which did not clearly favor AP1 or CRE sites (30). Although it is unclear which are the major Jun dimerization partners during regeneration, our data are compatible with Jun forming dimers with ATF3 (a known growth-promoting combination (30,50)), AP1 factors such as JUNB and FOSL2, but also BATF and the upregulated BATF3. Several other binding sites were identified in the promoter region analysis, notably MEF2 in the day 1 JunUP promoters, and MAF/MAFB, whose binding sites arise in the flanking regions of AP1 sites in these promoters. Both MAF forms are upregulated in a Jun-dependent manner. We hypothesize that these factors may co-operate with Jun in early regulation of RAG expression.

As well as acting via promoters, it is likely that Jun activity at enhancer sequences also contributes to regulation after axotomy. Indeed, a ChipSEQ study of Jun binding in sensory neurons showed changes in Jun binding at enhancers after axotomy (30). Regulation by Jun via enhancers may use different mechanisms and binding partners to those suggested by analysis of the promoter regions. However, the difficulty in linking specific genes to their enhancers precludes a similar analysis of these sequences.

### Functions of Jun targets

Jun target genes have a broad range of functions relevant for regeneration, including many signaling pathways and cell adhesion. Furthermore, Jun appears to have a general activating effect on the cell, increasing both metabolism and cytoskeleton production. Jun also appears to control the downregulation of neurotransmission machinery, such as post-synaptic components (including GABA receptors), which may be the gene expression correlate of synaptic stripping, and potassium channels, which may be related to the changes in electrical properties that occur post-axotomy (46,51). Some Jun targets recapitulate functions of Jun known in other cell types, such as control of cell death and the cell cycle. Regulation of these genes may be largely an unavoidable side effect of Jun activity, since if they are transcriptionally available there is limited scope for evolution to impose cell-type specific controls (52).

### Jun promotes regeneration over plasticity

Finally, as summarized in Figure 8f, a key role of Jun appears to be to direct the cell towards a regenerative response to axotomy rather than a plasticity-type response. The latter is a notable part of the early phase of the ‘alternative’ gene expression program occurring after axotomy in the absence of Jun and appears to invoke SRF as a key regulator. While the early regenerative response includes synaptic stripping, thought to be a mechanism to reduce excitotoxicity, this plasticity response involves the induction of synapse formation. Baseline synaptic density is reduced in KO motor neurons. This may result in reduced excitotoxic input when an axotomy occurs, so synaptic stripping may be unnecessary. However, by itself this does not explain why synaptic density increases in KO animals. Indeed, the trajectories of synapse density are quite opposite in KO and WT motor neurons. The lower baseline density suggests an imbalance in synapse homeostasis in the absence of Jun, although it is not clear what the mechanism may be here. At the same time, the reduced synapse density in uninjured neurons may allow the processes leading to synapse density increases to be more easily triggered.

The increase in synapse density is accompanied by expression of the plasticity-linked SRF target genes Fos, Egr1 and Egr2. Activation of SRF after axotomy is known to occur via phosphorylation of the cofactor Elk1, likely by axoplasmic ERK (53,54). However, normally the SRF target genes Fos and Egr1 are not upregulated in axotomized neurons (55). This pathway is linked to learning and plasticity, and in Jun KO mice appears to manifest as an increase in synapse density. In many neurons, particularly in the CNS, compensatory plasticity might be the appropriate response to the (partial) loss of an axon, explaining why this pathway is activated by axotomy. However, in a neuron that needs to regenerate, it appears that the SRF transcriptional response is suppressed and our data suggests Jun is critical to this suppression.

It is likely that, at least in the mammalian CNS, neurons have evolved to favor plasticity over axon regeneration by default, as this is an effective response to stroke and traumatic brain injury, and also given the importance of learning as a CNS function. Facial motor neurons, surprisingly, seem to express the pathway that leads to a plasticity response to axotomy, although it is normally latent. In this case, Jun, as well as coordinating a large part of the RAG program, appears to have the additional role of suppressing this pathway in favor of regeneration. It will be interesting to determine if the plasticity response would also manifest in other motor neuron types when Jun is knocked out, and in sensory neurons, which lie outside the CNS. Interestingly, the activation of SRF by axotomy was demonstrated in mouse sensory neurons (53). Overall, in injured facial motor neurons, Jun appears to boost the expression of the cell machinery for regeneration, and simultaneously suppress unwanted functions, including neurotransmission and plasticity, to favor axon regeneration.

In summary, in this work, we have fully defined the role of a specific TF in the control of the regeneration gene expression program in facial motor neurons. A similar approach applied to other factors, and the promoter analysis techniques employed here, will lead to a more complete understanding of the control of the RAG program, and thus enable development of rational TF-based strategies to promote axon regeneration in the injured CNS.

## Materials and Methods

### Animals

Homozygous floxed Jun mice (56) were crossed with mice expressing Cre recombinase under the Nestin promoter (57), as previously (22), and then bred and maintained as homozygous floxed-Jun/heterozygous nestin-Cre mice. Cre-positive mice have Jun deleted in the central nervous system and are referred to throughout as KO. Cre-negative offspring served as Jun-positive controls, referred to as WT. Male and female adult mice were used for experiments. Animals were kept on a 12 hour light/dark cycle and given food and water ad libitum.

### Surgery

All procedures were carried out in accordance with local animal experimentation rules. Animals operated in the UK were carried out with approval under the Scientific Procedures Act in the United Kingdom and in the Netherlands experimental procedures were approved by the local laboratory animal welfare committee and performed in accordance with European guidelines (2010/63/EU).

Animals that received facial nerve injury were anaesthetized with Avertin or isoflurane and the right facial nerve was exposed and cut near the stylomastoid foramen, taking care to cut also the retroauricular branch. The wound was closed with suture clips and the animals were allowed to recover in a heated chamber.

For gene expression profiling, a total of 37 animals were utilized. 29 animals were operated on. After 1 day (4 WT, 4 KO), 4 days (6 WT, 6 KO) and 14 days (5 WT, 4 KO), animals were anaesthetized with isoflurane and sacrificed. Brains were removed and frozen in OCT (Finetek) on dry ice. Brains of 8 uninjured animals (4 WT, 4 KO) were also obtained and frozen in the same way.

For validation of gene expression with histology and for quantification of synapse density, a further 30 animals were operated and 10 unoperated animals were sacrificed (n = 5 for each condition). At 1 day, 4 days or 14 days after surgery, animals were euthanized with pentobarbitone and perfused transcardially with PBS followed by 4% paraformaldehyde (PFA).

For experiments with adeno-associated viral vectors (AAV) expressing dominant negative SRF, 26 animals were used. Animals were injected with AAV6 expressing farnesylated GFP (fGFP) and dominant negative SRF or fGFP only. After allowing 2 weeks for transgene expression, animals either received a facial nerve injury (*n* = 7) or were sacrificed uninjured (*n* = 6). Animals were euthanized with pentobarbitone and perfused with PFA as before.

AAV delivery to the facial nucleus was carried out as follows. Mice were anaesthetized with isoflurane, an incision was made in the scalp along the midline, and the animal was mounted in a stereotaxic frame. The skull was positioned such that bregma and lambda were level to within 50 μm. The rostro-caudal position for injection was calculated as the midpoint of 1.75 mm caudal to bregma and 6.08 mm caudal to lambda. The other coordinates used for injection were 1.25 to the right of midline and to a depth of 6.05 mm. The distance from lambda to the caudal skull suture was measured and used to scale the coordinates appropriately, the standard measure being 7.75 mm. A hole was drilled in the skull, the dura mater opened with a fine needle and a glass needle containing AAV vector, mounted on the frame, inserted into the brain to the required depth. After a 3 min delay, 0.5 μL of viral vector (titer 1 × 10^12^ genomic copies/ml) was injected over 5 min. After a further 2 min delay, the needle was removed and the wound closed, post-operative analgesia was given, and the animals were allowed to recover.

### Laser dissection microscopy

Sections of brainstem were cut on a cryostat at 20 μm thickness onto PALM PEN–coated slides (Carl Zeiss b.v.), allowed to dry and stored at −80°C. To visualize the facial nuclei, slides were transferred to 70% alcohol containing 0.1% Cresyl Violet for 5 min at 4°C. Slides were then dehydrated in 100% EtOH at 4°C (2 × 5 min) and dried at room temperature. This allowed clear visualization of the facial nucleus, which was then excised using a PALM Laser Microbeam Microdissection (Carl Zeiss b.v.). RNA was extracted using Trizol and RNeasy microcolumns as described (58). RNA sample quality was assessed using an Agilent 2100 BioAnalyser (Agilent Technologies). RNA Integrity Numbers were in the range of 7.9 to 9.0.

### Gene expression profiling

Samples were amplified and labeled with the Agilent Low RNA Input Fluorescent Linear Amplification Kit (Agilent Technologies) using Cy3-CTP or Cy5-CTP (Perkin Elmer). Samples were hybridized to Agilent 44 k Mouse Whole Genome Arrays (part no. G4122F) and scanned using an Agilent DNA Microarray Scanner. Amplification, labeling and scanning were carried out as described (58). Expression profiles were analyzed using single channel intensity analysis, using the LIMMA package in R (59,60). Array channels were normalized first between and then within arrays using LIMMA with the ‘quantile’ method. Pairwise comparisons between groups were made using LIMMA. At each time point, WT animals were compared to KO animals, and expression profiles of WT animals at day 1, day 4 and day 14 were compared to those of the unoperated WT animals to determine the regulation after axotomy in WT animals. For all comparisons, a fold-change cut-off of 1.5 was used. A FDR, calculated using the Benjamini-Hochberg method (61), of 0.01 was used.

### Classification of genes and GO analysis

At each time point, all genes were classified first by their expression change in the WT animals following axotomy, as either upregulated (i.e. RAGs), downregulated or not regulated, compared to intact facial motor nuclei. Genes were then classified by the effect of Jun deletion on the expression profiles, as either higher in the KO, lower in the KO, or unchanged. Genes were then grouped as shown in Table 1. The resulting classes represent genes that are regulated after axotomy up or down in a Jun dependent manner (JunUP and JunDOWN) or are aberrantly up- or downregulated after axotomy in the absence of Jun (AltUP and AltDOWN). Genes in JunUP were further classified as completely Jun-dependent if no residual upregulation was observed in KO animals (using the fold change and FDR cut-offs given above), or partially Jun-dependent otherwise, i.e. genes which were still upregulated, but significantly less so than in WT animals. This classification was applied at each time point separately.

GO over-representation analysis was performed in R (60) with the TopGO package (62) using the ‘parentChild’ algorithm (63), and Fisher’s exact test. Annotations were downloaded from the Mouse Genome Informatics consortium (http://www.informatics.jax.org/) and the European Bioinformatics Institute (https://www.ebi.ac.uk/GOA/) and the two sources combined. The background gene list was taken as all genes with probes present on the array. Only over-represented GO classes with *P* < 0.05, an over-representation ratio > 2, and over 4 steps from the root node were considered. For a group with *n* genes classes with fewer than log_e_  *n* genes or fewer than 2 genes were discarded. The FDR was determined for each significant GO class in a gene list by random resampling of the background gene set and determining how many GO classes were found to be over-represented, meeting the criteria above, with a p-value lower than that of the GO class under test, taking the geometric mean of 100 trials. For presentation and classification, GO classes were grouped according to their parent–child relationships in the GO tree structure. Classification of GO classes into broader categories was performed manually.

### qPCR

cDNA for qPCR was made with random hexamer primers and MMLV Reverse Transcriptase (Invitrogen, Carlsbad, California). Quantitative PCR was carried out using 2x SYBR green mastermix (Applied Biosystems) and ABI 7300 real-time PCR system. qPCR primers used for validation are given in Supplementary Material, Table S2.

### Histological validation

Targets for validation of gene expression were chosen to represent over-represented GO functions in the JunUP class and genes in the AltUP class, including plasticity related TFs. Validation was performed at all time points with *n* = 2 or 3.

### ISH

Sequences used to generate probes for ISH are detailed in Supplementary Material, Table S1. These sequences were amplified by PCR from mouse cDNA and cloned into pBluescript SK. For 4 genes, the 60 bp sequence used in the microarray probe was used. To increase the signal of these short sequences, 700 bp of sequence of GAP43 mRNA in the sense orientation was included in the probe, 3′ to the specific sequence. For these probes, the control sense probe consisted of the 60 bp specific sequence in the sense orientation followed by the same GAP43 sense sequence. Digoxigenin-labeled sense and antisense riboprobes were generated using T7 and T3 RNA polymerases.

ISH was carried out as previously described (64), with the following modifications for fixed tissue. Sections were cut at 20 μm thickness onto SuperFrost plus slides (ThermoFisher) and allowed to dry overnight before being stored at −80°C. Sections were incubated for 30 min in 40 μg/ml proteinase K in PBS, followed by 10 min in 4% paraformaldehyde/PBS. The cited procedure was then followed from the triethanolamine step onwards, with 0.1% triton X100 added to the hybridization and prehybridization buffers. Hybridization temperatures for each probe are given in Supplementary Material, Table S1. Washes were carried out at 55°C. None of the sense probes gave a signal under the conditions used.

For combined localization of fGFP with ISH signal, sections were first coverslipped with PBS containing RNaseOUT (Promega) diluted 1:50 and 500 ng/ml ethidium bromide (as a Nissl stain), and fluorescent microscopy images were taken of GFP native fluorescence and the red Nissl stain. Coverslips were then removed and slides placed in cold 70% EtOH prior to ISH being carried out. Subsequently images of ISH and fluorescence were aligned with ImageJ (plug-in ‘Align Image by line ROI’).

### Immunohistochemistry

The following antibodies and final dilutions were used: rabbit anti-Fos (Santa Cruz, sc253, 1:4000), rabbit anti-Egr2 (Covance, PRB-236P, 1:1600); mouse anti-MAP2 (Chemicon MAB3418, 1:500), rabbit anti-synaptophysin (Abcam Ab14692, 1:200), chicken anti-GFP (Chemicon AB16901, 1:1000). All primary antibodies were incubated overnight, and all antibodies were diluted in IHC blocking medium (10% fetal calf serum, 1% bovine serum albumin, 0.1% Triton X100 in PBS) for incubation. Fos and Egr2 incubations were followed with biotinylated anti-rabbit (Vector labs, 1:200, 1.5 h), followed by ABC kit (Vector labs, 1:200, 1 h) and visualization with 3,3’-Diaminobenzidine (DAB) (0.025% DAB, 0.003% H2O2, 0.07% nickel ammonium sulphate in TBS).

MAP2 staining in combination with synaptophysin, Nissl staining and GFP was carried out as follows. To avoid cross-reactivity with endogenous immunoglobulins when using the mouse MAP2 antibody, this antibody was first incubated at 10× concentration with an F’ab fragment anti-mouse secondary antibody conjugated to DyeLight 649 (Jackson Immunoresearch Labs) at 1:50 in PBS. After 2 hours, mouse serum was added to 10% volume. After 1 more hour, the mixture was diluted 1:10 in IHC blocking medium, the synaptophysin and GFP antibodies added, and the mixture applied to the sections. After an overnight incubation and washes, the secondary antibodies anti-rabbit AlexaFluor594 and anti-chicken AlexaFluor 488 (both from Invitrogen; 1:600) were applied for 2 h.

For MAP2/synaptophysin/Nissl staining, ethidium bromide was applied at 500 ng/ml for 5 min to provide a red fluorescent Nissl stain (65), before coverslipping. For MAP2/synaptophysin/GFP/Nissl four-color staining, NeuroTrace Blue (Invitrogen) fluorescent Nissl stain was applied at 1:50 in PBS for 20 min.

### Microscopy

Images were acquired on a Zeiss Axioplan 2 microscope with a Retiga2000DC camera (fluorescence) or an Evolution MP color camera (brightfield), where a standard gamma correction was applied to color brightfield images, and on an Axioscan Z1 slide scanner.

### Quantification of gene expression in histological sections

Quantification of validation stainings (Fig. 4) was performed as follows. Areas of cytoplasm (ISH) or nuclei (immunohistochemistry for Fos and Egr2) of facial motor neurons and areas of background staining were manually labeled by a blinded experimenter. Staining intensities were quantified using ImageJ functionality, and the average background intensity was subtracted from neuronal intensities for each slide. Each slide always contained one WT and one KO section at the same time point, and the ratio of staining intensity of WT to KO was calculated within the slide for each pair of animals.

### A‌AV vectors

For AAV serotype testing in the facial nucleus AAV vectors packaged with pTR CGW (66) (containing GFP under a CMV promoter) was used, packaged in serotypes 2, 6, 7 and 8.

Dominant negative SRF (dnSRF) was generated by fusing the engrailed transcriptional repressor (67) to SRF. Amino acids 1–245 of human SRF (containing the DNA binding domain) was 3′ fused to amino acids 1–296 of Drosophila engrailed. SRF plasmid was obtained from David Ginty and the engrailed sequence was derived from a MEF2-En plasmid (68) obtained from Eric Olson. This was cloned into our previously described dual vector construct pAGLWFI (47), which contains back-to-back promoters and expresses farnesylated GFP on one side. DnSRF was cloned into the other expression slot. The resulting plasmid and pAGLWFI were packaged into AAV serotype 6. AAV production and titration were carried out as described (69). All viral titers were matched to 1 × 10^12^ genomic copies/ml for injection.

### Image processing

Seven training images of fluorescent Nissl-stained facial nuclei and 2 test images were manually segmented for facial motor neuron cell bodies and were used to train the U-NET neural network for image segmentation (44) using the ImageJ plugin as described in the tutorial. Training was carried out for 500 iterations with the learning rate of 0.1 and a further 500 with 0.01 learning rate. The resulting weights file was used to segment the Nissl stainings of facial nuclei. Subsequent analysis was carried out with scripts using ImageJ functionality. Motor neuron profiles were further filtered on size, and were kept if in the range 1500–5000 pixels. Outlines of the facial nuclei themselves were manually defined. Images of synaptophysin and MAP2 staining were processed with a band-pass filter and then thresholded with the Triangle automatic threshold function to obtain binary images. For motor neuron profiles, a 3-pixel rim of each motor neuron profile was taken for synapse quantification. Synaptophysin-positive and negative pixels were counted in each rim. For synapse quantification on dendrites, a 50-µm rim around each motor neuron profile was considered, and MAP2-positive areas only were quantified for synaptophysin staining as before.

Synapse density was expressed as a proportion of the area measured. Since proportions are not normally distributed, these were analyzed using quasibinomial general linear models using R (60). Where changes over time were being compared between WT and KO animals or viral vectors, a model including genotype, time and the interaction between the two or viral vector, time and the interaction was used.

### Promoter analysis

Transcription factor binding site (TFBS) position-frequency matrices were obtained from the CORE and PBM collections of JASPAR2020 (70) and from TRANSFAC public (71). Additional matrices for Jun dimers were derived from published SELEX data (72). Position weight matrices (PWMs) were derived from these by weighting for information content at each position (73,74). Each position in a promoter sequence can then be scored using the PWM, and a threshold applied to determine whether a site matches sufficiently. Because binding site motifs are generally short, there is a high occurrence of chance matches in any sequence for any given TFBS scoring matrix and threshold, most of which are non-functional. However, promoters regulated by a TF should have more matching sequences than non-regulated promoters, due to the presence of genuine functional binding sites. Therefore, we looked for over-representation of target sites in promoter regions of interest compared to a control set of unregulated genes. Promoter regions were defined as sequences up to 5 kb upstream of transcription start sites. Lengths of promoter regions analyzed were from 100 bp to 5 kb.

### Flexible threshold testing

Many algorithms are available for identification of TFBS in promoter sequences. Almost all algorithms employ fixed scoring thresholds to determine whether a sequence matches a given PWM, typically either the same fixed threshold for all PWMs, or a pre-calculated one for each PWM. For example, TRANSFAC offered thresholds for each PWM to maximize false positives, based on the presumed lack of functional binding sites in the second exon of coding mRNAs, or false negatives, or both (74). The score of a given sequence with a PWM is related to the binding affinity for the relevant TF. However, this affinity may vary depending on the presence of co-factors, and so the threshold between binding and non-binding may also vary depending on the biological states being compared.

An alternative approach, used by the F-match algorithm (75), is to optimize the threshold for each PWM for the given set of promoters to attain the maximum sensitivity (see below for how this is implemented). Although the resulting thresholds may then vary between datasets, this reflects the fact that binding affinity of a given TF for target sequences, and thus the optimum score to differentiate bound and non-bound sites, may vary between biological conditions.

A control promoter set was chosen of genes that showed no significant difference in expression due to Jun knockout and that also showed fold-change differences between genotypes of less than 1.3 fold. In addition, only genes with a maximum expression level over 7 (approximately the mean baseline expression level) were included, to exclude promoter regions, which might be in heterochromatin regions and thus inaccessible.

The following matrices were used to look for over-representation of AP1 and CRE sites in JunUP promoters: V$AP1_C from TRANSFAC; MA0018.3 (CREB1); MA0605.2 (ATF3); MA1632.1 (ATF2) (all from JASPAR).

### Threshold optimization

For each PWM representing a TF, scores at potential match sites were generated for the test promoter set and the control promoter set. Next, an optimized threshold was determined. This is achieved by compiling all the scores for a given PWM in the test and control promoter regions, to create the list of all possible thresholds, which are then tested exhaustively. For each possible threshold, the over-representation (OR) ratio, the ‘Additional Sites’ (AS) score, and the significance were calculated. Only scores were considered that gave a site frequency in the control promoter set of 0.01–0.2 sites/kilobase.

OR ratio was calculated as (A/lenA)/(B/lenB), where A is the number of matches in the promoter set under test, lenA is the total length of the promoters under test, B is the number of matches in the control promoter set, lenB is the total length of the control promoters. The ‘Additional Sites’ measure is calculated as A-(B*lenA/lenB).

Statistical significance of TFBS over-representation and under-representation was calculated with one-sided binomial tests, using the number of sites found in the test promoters and in the control promoters and the total respective lengths of each promoter set.

### Conserved score generation

To further improve sensitivity and specificity, we incorporated cross-species conservation in the scoring method. Functional binding sites are likely to be conserved by evolution, so much of the noise of false positives due to random occurrence of sequences can be removed by considering only conserved binding sites, a fact used by several available tools for identifying regulatory elements (76–78). Whole genome pairwise alignments of mouse genomes with those of rat, rabbit, guinea pig, human and marmoset were downloaded from UCSC (79) and used to create a map of each position in the mouse promoter sequences to its corresponding position in the other species. Mouse promoter sequences were first scanned for matches to each PWM with a low threshold (calculated to give a high frequency of matches). For each match in the mouse sequence, a central position in the match site was used for mapping to the other genomes. PWMs were scored on the aligned genomes at the mapped position with alignment gaps removed.

At each initial match in the mouse genome, the ‘conserved score’ was calculated as follows. If the requirement is that a site is conserved in *n* species, the scores across all the genomes are ranked in descending order and the *n*th score is taken. Whatever threshold is chosen, the site will meet the conservation requirement if and only if this conserved score is over the threshold. Thus, the multiple scores from the various species at each position are reduced to a single score at that position, and the algorithm can proceed as for the single species case, as described above.

### Grouping

Because many of these TFBS scoring matrices are similar, matrices were grouped if they identified sites in identical locations. If at least 50% of a given PWM’s hits were in the same location (within 4 bp) as those of another PWM they were grouped together.

### FDR

The FDR was calculated for binding site groups (group-wise FDR) for each analysis by a resampling method. For each resampling run, the analysis was carried out on randomized sets of genes drawn from the combined sets of test and control genes. If an analysis compared *a* promoters of interest to *b* control promoters, each test run compared *a* random promoters from the combined promoter set to the remaining *b* promoters. Over-represented sites were grouped by location as above, 100 test runs were performed and the average number of groups with an equal or better optimum value of the score under optimization was calculated. All reported results have a group-wise FDR < 0.05 unless otherwise stated.

### Flanking region analysis

Regions flanking 100 bp on either side of previously identified sites matching given PWMs were used, where all initial matching sites were excluded, and random 200 bp fragments from the control promoter set were used for the comparison.

Code for the analysis of promoter regions will be made available on request.

**Abbreviations**
AAV: adeno-associated vector
AS: additional sites
DAB: diaminobenzidine
DnSRF: Dominant negative SRF
fGFP: farnesylated GFP
GO: gene ontology
ISH: *in situ* hybridization
OR: over-representation
PFA: paraformaldehyde
PWM: position weight matrix
RAG TF: regeneration-associated transcription factor
RAG: regeneration-associated gene
SRF: serum response factor
TF: transcription factor
TFBS: transcription factor binding site
TSS: transcription start site

## Supplementary Material

Supplementary_Tables_and_Figs_ddab315

Supp_Data_1_ddab315

Supp_Data_2_ddab315

## Notes

Microarray data are deposited in the NCBI GEO database, accession no.: GSE162977.
