# Erratum to: The Jun-dependent axon regeneration gene program: Jun promotes regeneration over plasticity

**DOI:** 10.1093/hmg/ddac044

**Published:** 2022-02-15

**Authors:** Matthew R J Mason, Susan van Erp, Kim Wolzak, Axel Behrens, Gennadij Raivich, Joost Verhaagen

**Affiliations:** Laboratory for Regeneration of Sensorimotor Systems, The Netherlands Institute for Neuroscience, Royal Netherlands Academy of Arts and Sciences (KNAW), Meibergdreef 47, Amsterdam 1105BA, The Netherlands; Laboratory for Regeneration of Sensorimotor Systems, The Netherlands Institute for Neuroscience, Royal Netherlands Academy of Arts and Sciences (KNAW), Meibergdreef 47, Amsterdam 1105BA, The Netherlands; Laboratory for Regeneration of Sensorimotor Systems, The Netherlands Institute for Neuroscience, Royal Netherlands Academy of Arts and Sciences (KNAW), Meibergdreef 47, Amsterdam 1105BA, The Netherlands; Cancer Stem Cell Laboratory, The Institute of Cancer Research, 237 Fulham Road, London SW3 6JB, UK; Department of Surgery and Cancer, Imperial College London, London SW7 2AZ, UK; Convergence Science Centre, Imperial College, London, SW7 2BU, UK; UCL Institute for Women's Health, Maternal and Fetal Medicine, Perinatal Brain Repair Group, London WC1E 6HX, UK; Laboratory for Regeneration of Sensorimotor Systems, The Netherlands Institute for Neuroscience, Royal Netherlands Academy of Arts and Sciences (KNAW), Meibergdreef 47, Amsterdam 1105BA, The Netherlands; Center for Neurogenomics and Cognition Research, Neuroscience Campus Amsterdam, Vrije Universiteit Amsterdam, Amsterdam 1081HV, The Netherlands

In the originally published version of this manuscript, there were errors in the details and enumeration for authors' affiliations. These should read:

“Matthew R J Mason^1^, Susan van Erp^1^, Kim Wolzak^1^, Axel Behrens^2^, Gennadij Raivich^3^, Joost Verhaagen^1,4^

1 Laboratory for Regeneration of Sensorimotor Systems, The Netherlands Institute for Neuroscience, Royal Netherlands Academy of Arts and Sciences (KNAW), Meibergdreef 47, Amsterdam 1105BA, The Netherlands

2 Cancer Stem Cell Laboratory, The Institute of Cancer Research, 237 Fulham Road, London SW3 6JB, UK; Department of Surgery and Cancer, Imperial College London, London SW7 2AZ, UK; Convergence Science Centre, Imperial College, London, SW7 2BU, UK

3 UCL Institute for Women's Health, Maternal and Fetal Medicine, Perinatal Brain Repair Group, London WC1E 6HX, UK

4 Center for Neurogenomics and Cognition Research, Neuroscience Campus Amsterdam, Vrije Universiteit Amsterdam, Amsterdam 1081HV, The Netherlands” instead of

“Matthew R.J. Mason^1^, Susan van Erp^1^, Kim Wolzak^1^, Axel Behrens^1,2,3^, Gennadij Raivich^3^ and Joost Verhaagen^1,4^

1 Cancer Stem Cell Laboratory, The Institute of Cancer Research, 237 Fulham Road, London SW3 6JB, UK

2 Department of Surgery and Cancer, Imperial College London, London SW7 2AZ, UK

3 Convergence Science Centre, Imperial College, London, SW7 2BU, UK

4 Center for Neurogenomics and Cognition Research, Neuroscience Campus Amsterdam, Vrije Universiteit Amsterdam, Amsterdam 1081HV, The Netherlands”. These errors have now been corrected in the article online.

## Notes

Microarray data are deposited in the NCBI GEO database, accession no.: GSE162977.

