# Supplementary material for: The Jun-dependent axon regeneration gene program: Jun promotes regeneration over plasticity
Source: Hum Mol Genet. 2021 Oct 28;31(8):1242–62. doi: 10.1093/hmg/ddab315 (PMC9029231; doi:10.1093/hmg/ddab315)
Supplement: Supplementary_Tables_and_Figs_ddab315 [file supplementary_tables_and_figs_ddab315.docx]

| *gene* | *Genbank accession nr.* | *start* | *end* | *length (bases)* | *hybridisation temperature (°C)* |
| --- | --- | --- | --- | --- | --- |
| Casp6 | NM_009811.4 | 165 | 950 | 786 | 60 |
| Ccdc68 | NM_201362.2 | 59 | 851 | 793 | 55 |
| Fgf3 | NM_008007.2 | 464 | 1185 | 722 | 65 |
| Flrt3 | NM_178382.4 | 1542 | 2501 | 960 | 55 |
| Msh6 | NM_010830.2 | 3198 | 4086 | 889 | 55 |
| Npy | NM_023456.3 | 94 | 466 | 373 | 60 |
| Nrap | NM_198059.3 | 827 | 1596 | 770 | 55 |
| Pop5 | NM_026398.4 | 428 | 960 | 533 | 65 |
| Rras2 | NM_025846.2 | 1081 | 1836 | 756 | 55 |
| Shh | NM_009170.3 | 462 | 1109 | 648 | 65 |
| Speer | NM_025759.3 | 77 | 910 | 834 | 65 |
| Sprr1a | NM_009264.2 | 7 | 693 | 687 | 65 |
|  |  |  |  |  |  |
| *gene* | *Agilent probe ID of sequence used* | | | |  |
| Egr1 | A_51_P367866 | | | | 55 |
| Fgf21 | A_51_P315925 | | | | 58 |
| Gdf15 | A_52_P532982 | | | | 55 |
| Reg3b | A_51_P169671 | | | | 58 |

**Supplementary Material**

**Supplementary Tables**

Table S1. Sequences used to generate riboprobes for in situ hybridization. Where GenBank IDs are given, sequences were generated for partial cDNA sequences corresponding to the sequences indicated by RT-PCR. Where Agilent probe IDs are given, 60-base sequences identical to the microarray probes sequences were cloned and used to make riboprobes.

| *gene* | *forward primer* | *reverse primer* |
| --- | --- | --- |
| 9230110K08Rik | GAGCTCCAGATCCTCTCTG | GACTGGCTTTGACGATCAAT |
| Adam8 | CGAGTGCTGGAGGTTGTA | GACAAGAAGTTCTCCAGTGT |
| Adcyap1 | CCAGCTCCAGACAGTGA | TCCGCTACACATGGTCATT |
| Aldh1l1 | GAAGAGCTAGCCACCATT | GGAAGGTCTGGATGGACA |
| Bdnf | GGATGAGGACCAGAAGGTT | TAGACATGTTTGCGGCATC |
| Casp6 | CAGTACAAGATGGACCACAA | GAACCTCTCGTGATTGAAGAT |
| Crem | TGGGTGCCACAGTTGAA | AGTACAAAGAGCAACCAACA |
| Egr1 | GATCCCTGACTATCTGTTTCC | TAGTGGATAGTGGAGTGAGC |
| Eno2 | CGAAGTACAACCAGCTCAT | TCACAGCACACTGGGATT |
| Fgf21 | ACCGCAGTCCAGAAAGT | CAAAGTGAGGCGATCCATAG |
| Flrt3 | ACAACTCCATCCTGGAAATC | TGTACAGATTCATCCCATTCG |
| Fos | AGCCTTTCCTACTACCATTC | CAAAAGTCCTGTGTGTTGAC |
| Gdf15 | GGACTCGAACTCAGAACCA | CACGCGGTAGGCTTC |
| Mobp | CAAGAACCAGAAGTTCTCCG | TCTCACGCTTGGAGTTGAG |
| Msh6 | GATTATGACCAAGCACTTGC | CTTCAGGTAGATTACGGGTAG |
| Nrap | CAGCAACCAGATCACCAAT | ACCAAGGGTCATCGTGTA |
| Pop5 | GATTTCTACCAGCTCGTGT | ATCAGGAACTTCTGACAGGT |
| Rras2 | TCTCTGCTACCGTTTACATC | AGACCGTTACATTAGCTTCAC |
| Shh | TCCGAACGATTTAAGGAACTC | CTTTGCACCTCTGAGTCATC |
| Slc5a7 | CCAGCTTTCCTTCAGACAAA | CAGAGCCCATACACAGTCTT |
| Sprr1a | TTATGAGTCCCATTGCCTTG | CCCATTCAGATGAATCCTGAG |
| Srf | GTTCAGCTTCACCAGATGG | ATTCACTCTTGGTGCTGTG |
| Wnt7a | AAGTGGACTGCTTGAGGT | CCCAGTCCTAGCAAGTCA |

Table S2. Primer sequences used for qPCR confirmation of gene expression profiles.

**Supplementary Figures**

**
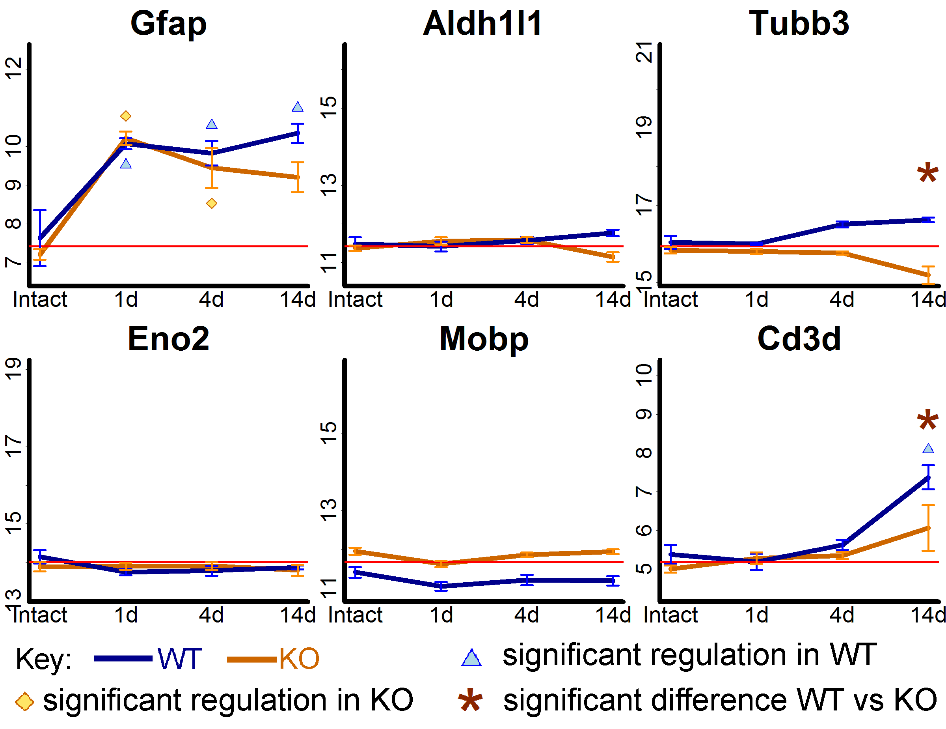
**

Figure S1. Expression of cell-type specific markers in WT and KO facial motor nuclei in uninjured animals and after axotomy. No significant baseline differences are seen indicating that the cellular makeup of the facial nucleus is largely unchanged. Gene expression is shown on a log_2_ relative scale as mean ± SEM.


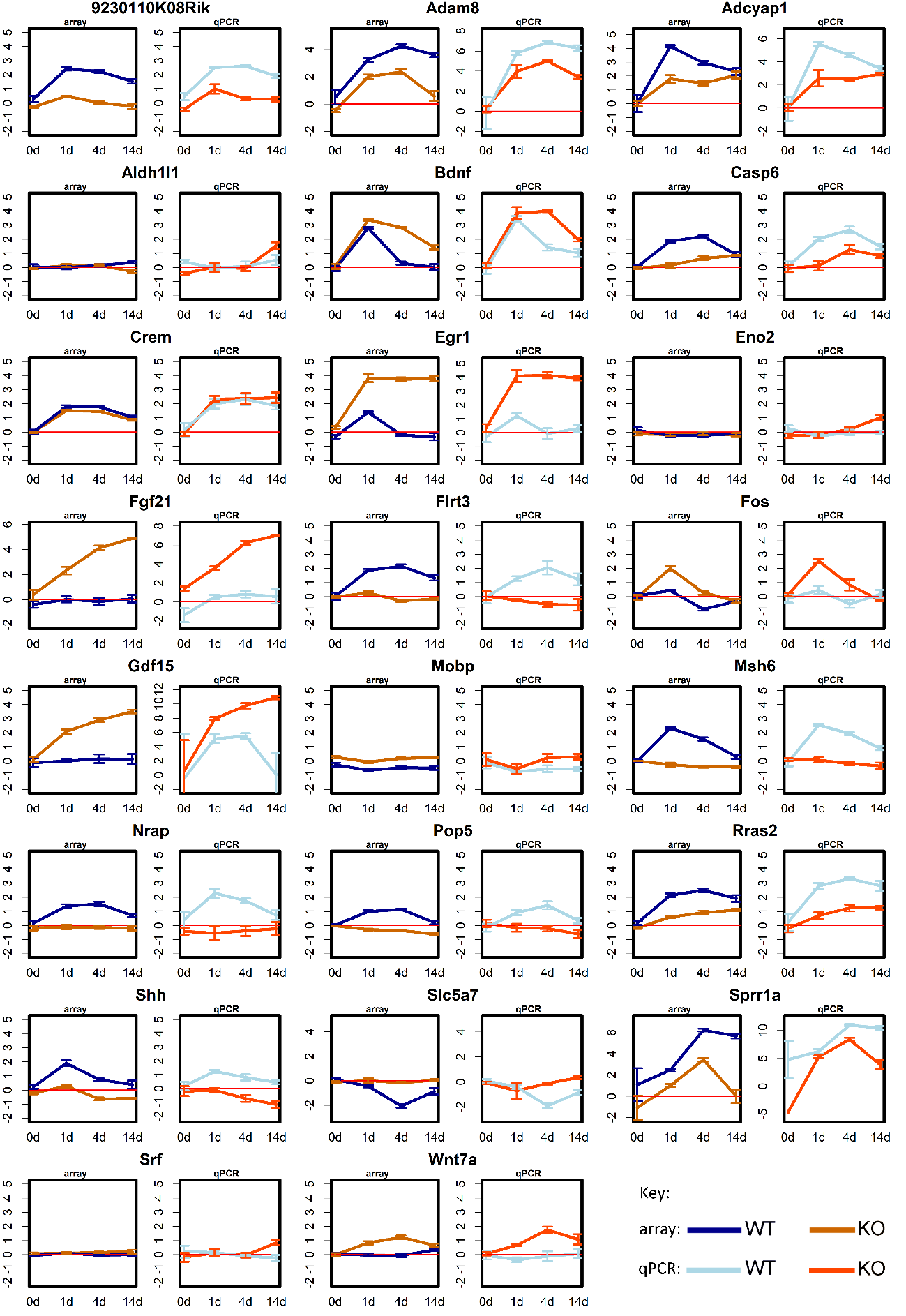
Figure S2. Validation of gene expression profiles by quantitative PCR (qPCR). Each pair of panels shows expression profiles as determined by the microarray (left) and by qPCR (right). In almost all cases the expression profiles are in good agreement. Genes were selected that belong to JunUP or AltUP categories, or are known RAGs, or are cell-type markers, with the addition of SRF which was included because of its relevance to the AltUP program (see Results). Gene expression is shown on a log_2_ relative scale as mean ± SEM.


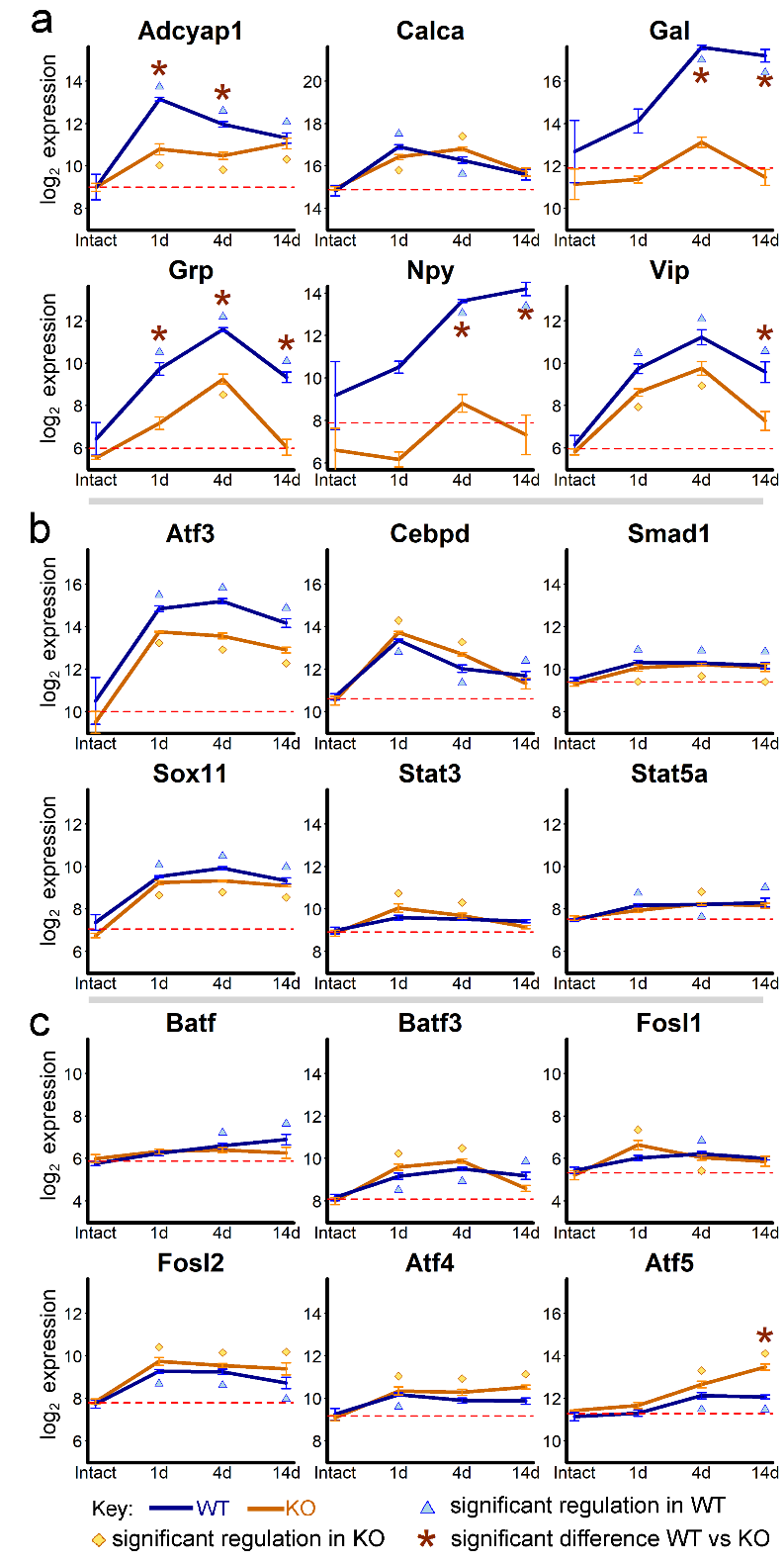


Figure S3. Example expression profiles of three categories of genes. (a) Neuropeptides. Several neuropeptides (Adcyap1, Grp, Npy, Gal) are dependent on Jun for their upregulation after axotomy, although Calca (CGRP) is not and VIP only at 14 days. (b) Known RAG TFs that are independent of Jun. This includes ATF3, a known binding partner of Jun. (c) Other transcription factors of the AP1 family which are regulated in WT animals after axotomy but for the most part are unaffected by Jun deletion. Gene expression is shown on a log_2_ relative scale as mean ± SEM.


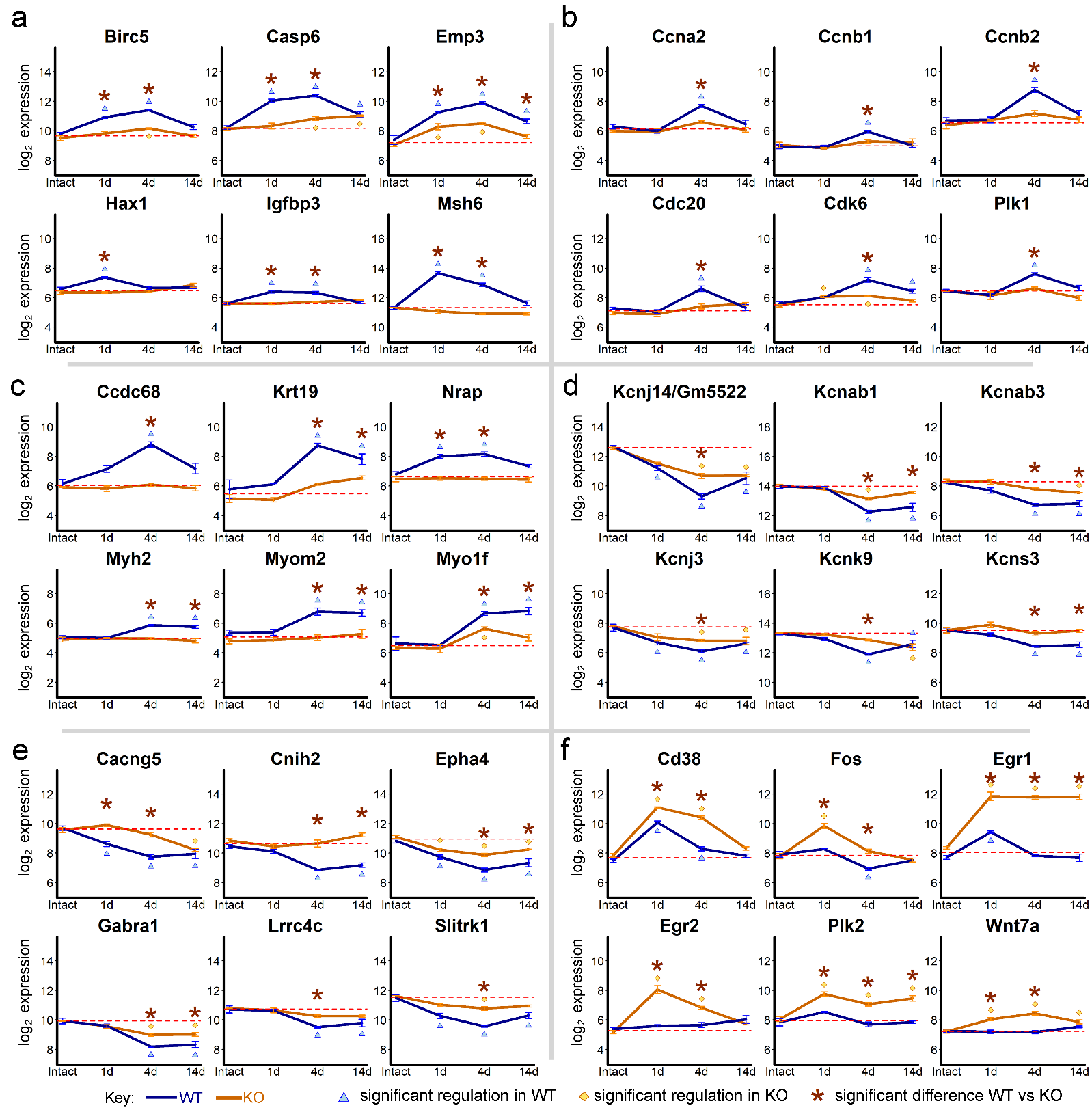


Figure S4. Example expression profiles of six categories of genes identified by Gene Ontology analysis. (a) Apoptosis linked genes in the JunUP category. (b) Genes involved in cell-cycle control, found in the JunUP category at day 4. (c) Genes related to actin cytoskeleton organisation, potentially important for regeneration, which are in the JunUP category. (d) Voltage-gated potassium channels and in (e), post-synaptic cell components which are both in the JunDOWN category and indicate Jun-dependent suppression of neurotransmission machinery. (f) Genes in the AltUP category linked to synaptic plasticity and learning and memory. Gene expression is shown on a log_2_ scale as mean ± SEM.


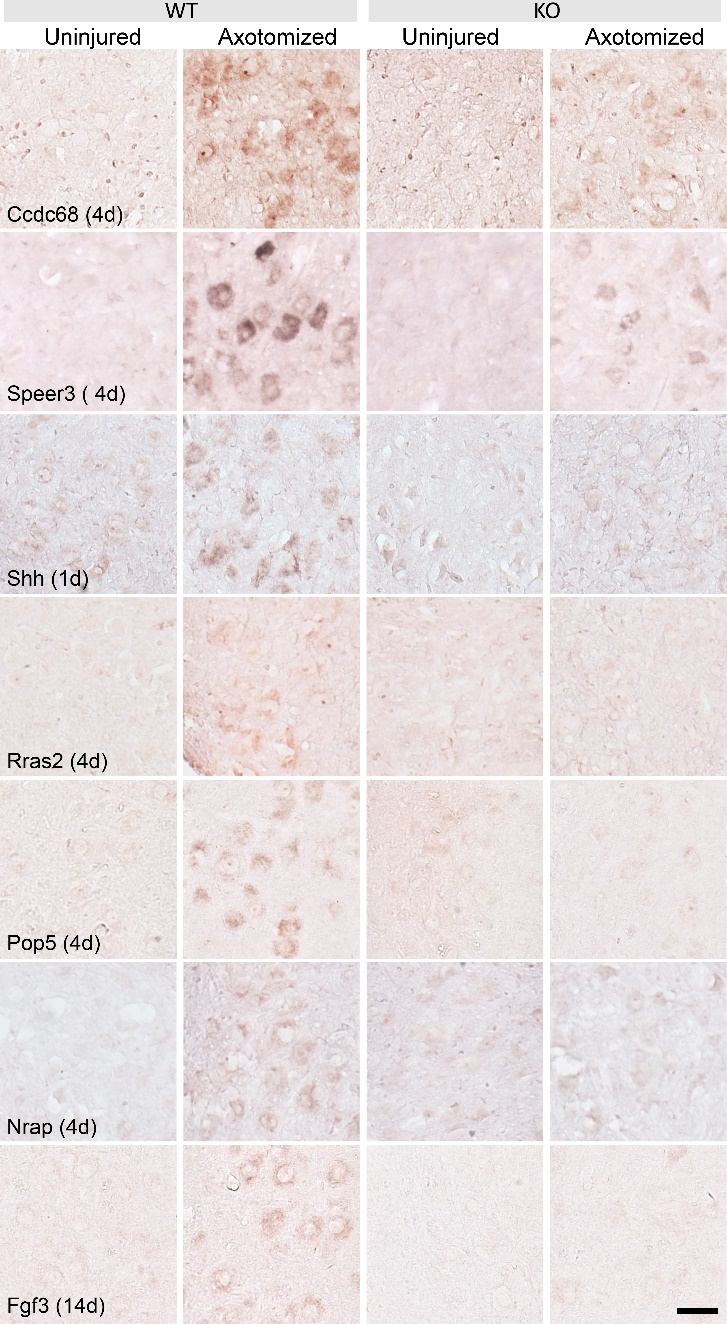


Figure S5. Histological confirmation and localization of gene expression by in situ hybridisation (ISH). Time points after axotomy are indicated by the labels. Many of these genes are novel RAGs and were selected because they show Jun-dependent upregulation after axotomy. ISH shows that all targets are regulated as expected and upregulation in the facial nucleus is confined to the motor neurons. Scale bar 50µm.


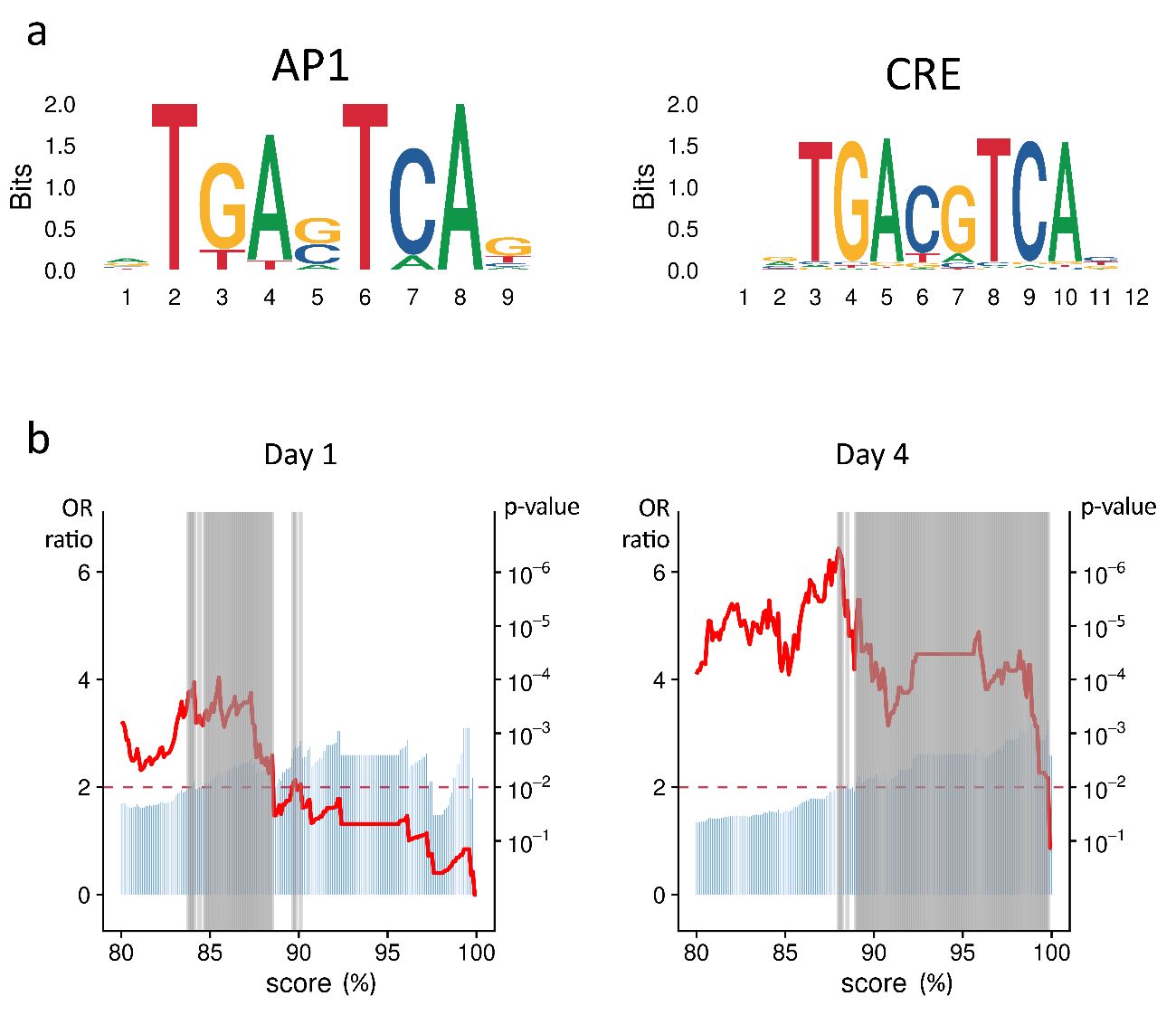


Figure S6. Promoter analysis binding site motifs and the effect of varying the scoring threshold on binding site over-representation. (a) Sequence logos for the two major types of biding site that may be used by Jun: an AP1 site and a CRE site. Note that The CRE site is essentially similar to the AP1 site with the insertion of a single base at the core of the sequence motif. Logos are derived from the TRANSFAC matrix V$AP1_C and the JASPAR matrix MA0018.3 (CREB1). (b) This shows the effect of varying the scoring threshold on AP1-site over-representation in the JunUP promoters, using the TRANSFAC V$AP1_C matrix. The x-axis shows the scoring threshold applied. The blue bars depict the over-representation ratio of sites in JunUP promoters to sites in control promoters (left y-axis) while the red line shows the p-value (binomial test; right y-axis). The grey shading indicates where the relatively modest thresholds of 2-fold over-representation and p-value of 0.01, are exceeded. Significant over-representation (OR) is seen only at a subset of thresholds in the Day 1 JunUP promoters (left). However looking at Day 4 JunUP promoters (right), OR is seen at a set of thresholds that is almost mutually exclusive with those where OR is found at Day 1. Optimising the threshold for each promoter set therefore will improve sensitivity.


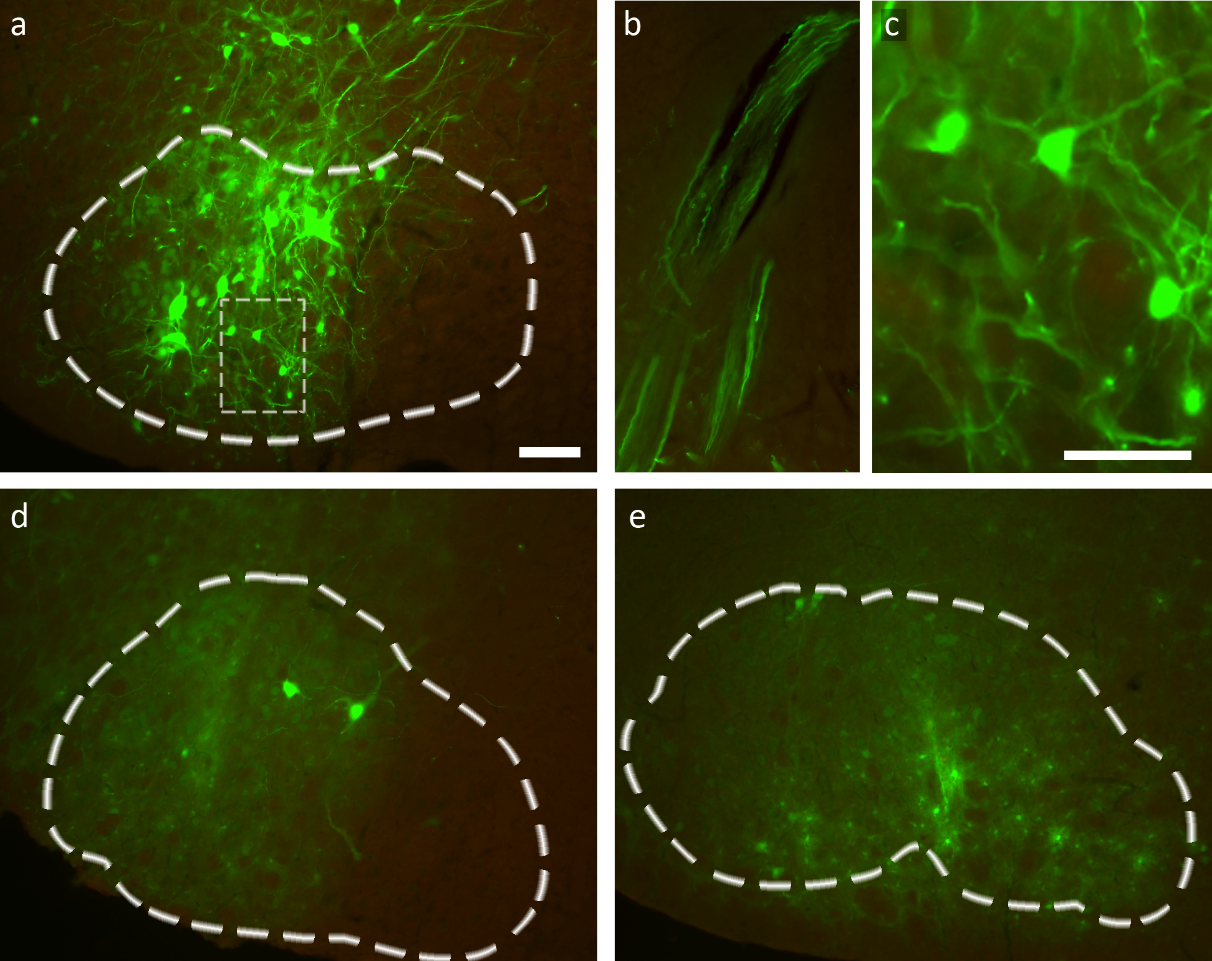


Figure S7. Testing of AAV serotypes 6, 7 and 8 in the mouse facial nucleus. AAV2 was also tested but gave no detectable expression. Each serotype contained an expression cassette expressing GFP. 5x10^8^ genomic copies of each virus in 0.5μl was injected into the right facial nucleus (a) AAV6 shows clear and strong neuronal expression. Scale bar: 100μm, applies to (a,b,d,e). (b) GFP-positive fibres can be seen in the facial nerve as it runs through the brainstem rostral to the facial nucleus. (c) Higher magnification of the inset in (a) Scale bar 50μm. (d) AAV8. Expression is neuronal but weaker than that from AAV6. (e) AAV7. Some neuronal expression is seen but many smaller cells that appear morphologically to be astrocytes also express GFP.


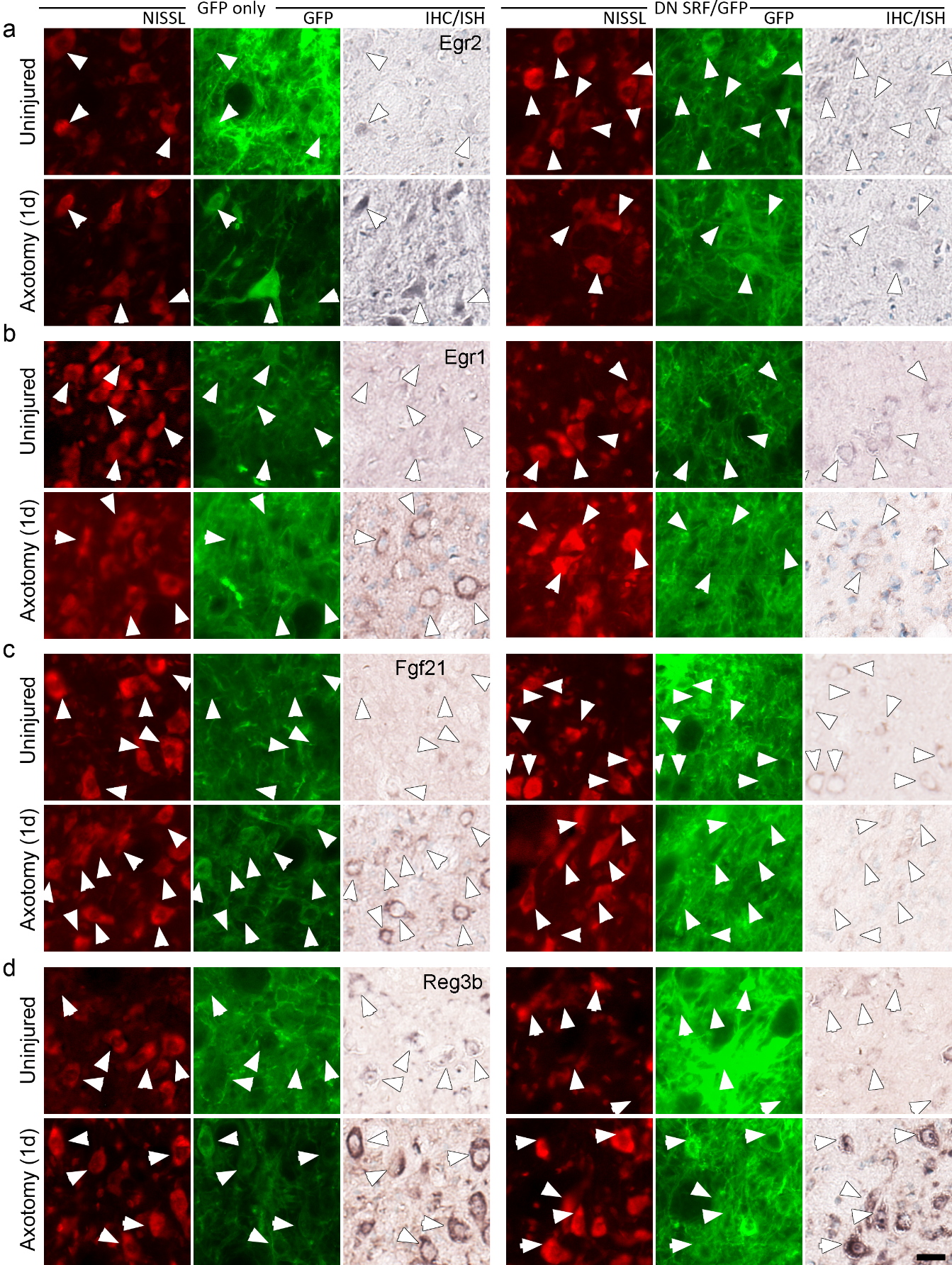
Figure S8. Dominant SRF prevents upregulation of several more genes in the AltUP program (genes that are more or *de novo* upregulated after axotomy in KO animals). Arrows indicate motor neurons visible in Nissl staining that are GFP positive and thus transduced. (a)-(d) show immunohistochemistry or in situ hybridisation in KO animals in neurons expressing fGFP and DN SRF, or fGFP only. (a) IHC for Egr2. (b) ISH for Egr1. (c) ISH for Fgf21. (d) ISH for Reg3b. In (a-c) the left set of six panels shows induction of expression by axotomy in motor neurons expressing fGFP only, while this is blocked in neurons expressing DN SRF (right six panels). (d) However in the case of Reg3b, expression still increases in neurons expressing DN-SRF (see also Figure 8). Scale bar 25 μm.
